# A mixed methods systematic review of digital interventions to support the psychological health and well-being of people living with dermatological conditions

**DOI:** 10.3389/fmed.2022.1024879

**Published:** 2022-11-03

**Authors:** Rachael M. Hewitt, Matthew Ploszajski, Catherine Purcell, Rachael Pattinson, Bethan Jones, Georgina H. Wren, Olivia Hughes, Matthew J. Ridd, Andrew R. Thompson, Chris Bundy

**Affiliations:** ^1^School of Healthcare Sciences, Cardiff University, Cardiff, United Kingdom; ^2^Wales Centre for Evidence Based Care–A JBI Centre of Excellence, Cardiff, United Kingdom; ^3^School of Psychology, Cardiff University, Cardiff, United Kingdom; ^4^School of Health and Social Wellbeing, University of the West of England, Bristol, United Kingdom; ^5^Population Health Sciences, University of Bristol, Bristol, United Kingdom; ^6^South Wales Clinical Psychology Training Programme, Cardiff and Vale University Health Board – School of Psychology, Cardiff University, Cardiff, United Kingdom

**Keywords:** systematic review, dermatology, psychology, digital health, behaviour change

## Abstract

**Background:**

Dermatological conditions can have a substantial impact on psychological as well as physical health yet dedicated face-to-face psychological support for patients is lacking. Thus, individuals may require additional support to self-manage dermatological conditions effectively. Digital technology can contribute to long-term condition management, but knowledge of the effectiveness of digital interventions addressing psychological (cognitive, emotional, and behavioural) aspects of dermatological conditions is limited.

**Objectives:**

To identify, determine the effectiveness, and explore people’s views and experiences of digital interventions supporting the psychological health of people with dermatological conditions.

**Methods:**

A mixed methods systematic review informed by JBI methodology. The protocol was registered on PROSPERO. Eight electronic databases were searched for papers written between January 2002 and October 2021. Data screening and extraction were conducted in Covidence. The methodological quality of studies were scrutinised against JBI critical appraisal tools. Intervention characteristics were captured using the Template for Intervention Description and Replication checklist and guide. Data were synthesised using a convergent segregated approach. The results were reported in a narrative summary.

**Results:**

Twenty-three papers were identified from 4,883 references, including 15 randomised controlled trials. Nineteen interventions were condition-specific, 13 were delivered online, 16 involved an educational component, and 7 endorsed established, evidence-based therapeutic approaches. Improvements in knowledge, mood, quality of life, the therapeutic relationship, and reduced disease severity in the short to medium term, were reported, although there was substantial heterogeneity within the literature. Thirteen studies captured feedback from users, who considered various digital interventions as convenient and helpful for improving knowledge, emotion regulation, and personal control, but technical and individual barriers to use were reported. Use of established qualitative methodologies was limited and, in some cases, poorly reported.

**Conclusion:**

Some web-based digital psychological interventions seem to be acceptable to people living with mainly psoriasis and eczema. Whilst some digital interventions benefitted cognitive and emotional factors, heterogeneity and inconsistencies in the literature meant definitive statements about their effectiveness could not be drawn. Interdisciplinary and patient-centred approaches to research are needed to develop and test quality digital interventions supporting the psychological health of adults living with common and rare dermatological conditions.

**Systematic review registration:**

[https://www.crd.york.ac.uk/PROSPERO/display_record.php?RecordID=285435], identifier [CRD42021285435].

## Introduction

Dermatological conditions can impact all aspects of life with people commonly reporting psychological, social, financial, occupational, and educational consequences, plus challenges to daily activities, in addition to their physical manifestations (1–7, 8). Many individuals living with dermatological conditions consider the psychological impact to be most profound (2). In a recent survey of 544 people in the United Kingdom with a skin condition, 97.61% revealed that their emotional wellbeing had been negatively affected as a result of the condition (4). Impaired quality of life (QoL) and a range of mental health issues are recognised in people with dermatological conditions, across the spectrum of psychological conditions, including low mood, anxiety and depression, to suicidality (2) and psychoses (9). Inter-disciplinary and whole person approaches are, therefore, essential for condition management and improving QoL in people with dermatological conditions (7, 10, 11).

The 2013 All-Party Parliamentary Group on Skin report called for more integrated and dedicated psychological support within dermatology (10). The most recent iteration showed little positive change over the previous decade, as the provision of specialist psychological support within dermatology settings, and dedicated psychodermatology services, both remain limited (7). In addition, previous research has shown that dermatology staff report lacking confidence in their ability to address the psychological impact of dermatological conditions (12, 13) and that some dermatologists still fail to recognise (14) and manage dermatological conditions as long-term conditions (15). Thus, inadequacies in education and training for healthcare professionals on the psychological aspects of dermatological conditions persist (7, 10).

Many people with dermatological conditions report not being able to access psychological services (4), or being dismissed (2) by medical professionals who fail to understand (4), or even acknowledge (6) the severity of the psychological impact of dermatological conditions. Individuals report dissatisfaction with the quality of care leaving them feeling unsupported and with no choice but to cope with their condition alone (10, 8). Clearly, additional forms of support are needed to help people to live well with dermatological conditions (16).

Digital technology has transformed healthcare delivery (17), including dermatology (18). For example, both asynchronous and synchronous teledermatology is now widely embedded within dermatology service provision (18), yet the primary focus has been on the assessment, diagnoses, and monitoring of physical symptoms and treatments (18, 19), with little to no consideration given to the psychological impact of that condition on the individual.

Interventions using digital technology, including the internet and smartphone applications (apps), have proved to be effective in facilitating the management of other long-term conditions (17). For example, people living with type 2 diabetes (20) and cancers (21) consider them a useful and convenient adjunct to standard care that inform, enable and empower individuals to control their health and lifestyle (22). In the context of dermatology, digital health interventions are limited; some have been developed mainly for skin cancer, focusing on primary prevention (23, 24). Digital technology could provide a platform for delivering psychological support to adults with dermatological conditions, but it is not clear what works or what delivery methods are acceptable to this group.

We conducted a mixed methods systematic review to identify existing digital programmes, determine their effectiveness, and explore people’s views and experiences of available programmes for supporting the psychological health and well-being of adults living with dermatological conditions.

## Methods

The present systematic review was informed by the JBI methodology for conducting mixed method systematic reviews (25).

### Eligibility criteria

We developed comprehensive inclusion and exclusion criteria to judge the eligibility of papers for inclusion in this systematic review. The criteria were developed *a priori* based on the results of a preliminary scoping search on the MEDLINE (Ovid) database and were piloted on three papers identified through the initial search. The eligibility criteria were independently applied by RH and one other reviewer (GW or OH). The reviewers discussed potential changes and the eligibility criteria were updated prior to application. The full eligibility criteria are outlined below.

#### Study design

Qualitative, quantitative, and mixed methods studies written in English were included. Systematic reviews, meta-analyses, study and review protocols, commentaries, editorials, grey literature, conference posters, abstracts, and papers on intervention development, were excluded.

#### Participants

We included studies concerning adults (18+ years) with a clinician- or self-diagnosed dermatological condition, either with or without established comorbidities. Papers focused on children and adolescents, or people with non-dermatological conditions or mental, psychological, psychiatric disorders only, were excluded.

#### Interventions

Eligible interventions were those designed for patient use, delivered by digital technology, accessed online or offline, and comprised of at least one of the following interactive components:

• Patient-to-patient communication.
• Patient-to-practitioner communication.
• On-demand information services.
• Personal health tracking.
• Targeted communication.

This definition of digital interventions was adapted from an existing definition (26), which was based on the World Health Organization’s classification (27). We extended the existing definition to encompass The Medical Research Council’s definition of complex interventions (28).

Digital interventions for detecting, diagnosing, triaging, or assessing physical symptoms, asynchronous telemedicine, and psychological interventions delivered via telephone or email, were not included in this review.

#### Comparators

Eligible comparators included none or alternative intervention and standard care.

#### Outcomes

We prioritised psychological outcomes (cognitive, emotional, and behavioural) and considered other outcomes if they were measured alongside a psychological outcome(s). A non-exhaustive list of examples of eligible outcomes are presented in Table 1.

**TABLE 1:** Examples of primary and secondary outcomes.

Category	Examples
**Primary outcomes**	
Cognitive	Beliefs about illness, beliefs about treatment, knowledge
Emotional	Fear, stress
Behavioural/behaviour change	Diet and weight management, physical activity or exercise, smoking, alcohol consumption, sleep, medication adherence
Other psychological	Adjustment, self-efficacy, self-compassion, motivation, quality of life, health-related quality of life, depression, anxiety
Secondary outcomes*	
Physical	Pain, severity, duration, skin coverage
Usage data metrics	Number of log ins, modules accessed, time spent on/using intervention
Other	Intervention feasibility, acceptability or usability, user satisfaction or engagement

*Only included if measured in addition to at least one psychological outcome.

### Systematic review protocol

The review protocol was registered on PROSPERO in October 2021 (reference number: CRD42021285435).

### Search strategy

We ran a preliminary search of MEDLINE (Ovid) on 15th October 2021 to scope the existing literature on the review questions. The scoping exercise helped to ensure there were no current or ongoing reviews on the topic, to refine the aims and eligibility criteria for this systematic review, and to estimate the amount of published work available and, therefore, the resources needed to complete this systematic review. Relevant papers identified from a scoping search of MEDLINE were also used to develop a full search strategy; key words in the titles and abstracts, and the index terms used to describe the papers, were organised into search strings with support from a specialist subject librarian (see Supplementary material, section 1).

The search period spanned 1st January 2002 to 29th October 2021. We only included papers published from 2002 onwards because this year followed the publication of an influential paper on defining eHealth (29), which marked the beginning of a global increase in the implementation of eHealth policy and strategies (30).

### Data sources

We searched the following electronic databases for peer-reviewed material:

• MEDLINE, EMBASE, Emcare, PsycINFO (Ovid).
• CINAHL (EBSCO).
• Scopus.
• Web of Science.

We also conducted a search of the Open Science Framework Preprint Archive for unpublished papers, but no papers relevant to the review questions were retrieved.

### Article screening

References were imported into EndNote X9 (Clarivate Analytics USA), and duplicates were removed. References were subsequently imported to Covidence; an online platform designed to support the conduct of systematic reviews. More potential duplicates were identified automatically in Covidence, which were reviewed and later removed by the review team.

A two-step screening process determined the papers included for analysis. Firstly, titles and abstracts of papers were screened against the eligibility criteria. All were screened independently by RH and one other reviewer (MP, BJ, RP, GW, or OH) using a screening tool developed for the purpose of this systematic review (see Supplementary material, section 2). Any conflicts that arose were resolved by a third reviewer (CP, MR, or AT).

The full texts of the remaining papers were screened independently by RH and another reviewer (MP, BJ, RP, GW, or OH), using the screening tool. The reference lists of full texts were also screened to ensure no potentially relevant papers had been missed. Reasons for exclusion were recorded and one reviewer (RP) was responsible for resolving disagreements at this second stage.

The screening process was reported in the Preferred Reporting Items for Systematic Reviews and Meta-analyses (PRISMA) 2020 flow diagram (31).

### Data extraction

Data were independently extracted in Covidence by RH and another reviewer (MP, RP, BJ, GW, or OH). The research team conducted consensus checks and resolved discrepancies through discussion. Intervention characteristics were charted against the Template for Intervention Description and Replication (TIDieR) checklist and guide (32), which we adapted to capture for *whom* interventions were intended. Specific intervention features were captured independently by RH and another reviewer (MP, RP, BJ, GW, or OH) before discrepancies were resolved through team discussion.

### Critical appraisal

We assessed the methodological quality of included papers using established JBI critical appraisal tools for the following study designs: Randomised Controlled Trials (RCTs) and quasi-experimental studies (33); analytical cross-sectional studies, case reports, and cohort studies (34); and qualitative research (35).

We adopted the method outlined by Edwards and colleagues (36) to judge quality, and included studies were assessed against the pre-determined criteria. Quantitative and qualitative components of mixed methods studies were appraised separately using the appropriate critical appraisal instruments. Each paper received an overall score based on the number of criteria met (13 for RCTs, 10 for qualitative and cohort studies, 9 for quasi-experimental studies, 8 for analytical cross-sectional studies and case reports). Studies scored one for each criterion met and zero for any criterion for which the evidence was unclear. If a criterion was considered not applicable to a particular study, a point was deducted from the overall score; for example, if the total possible score was 10, one was deducted reducing the total possible to 9.

Each paper was assessed independently by RH and another reviewer (MP, RP, BJ, GW, or OH) and all scores were checked by a third reviewer. For completeness data were extracted from all papers irrespective of their quality score. In addition, each paper was also assigned to a JBI level of evidence for effectiveness (1 = high, 2 = moderate, 3 = low, 4 = very low) or meaningfulness (1–5), based on the study design reported (37). The purpose was to support healthcare professionals and others working in this area to form preliminary judgements of the rigour of the evidence presented in this review, and facilitate the implementation of quality evidence-based research in clinical and health settings (37).

### Data analysis

Papers were imported into NVivo 12 Pro where one reviewer (RH) conducted a content analysis to synthesise the data. This involved assigning codes to parts of the text which captured study and intervention characteristics and results relating to the main aims. The results of the content analysis were verified by two reviewers (CP and CB). The code book is included as Supplementary material (section 3).

One reviewer (RH) employed a convergent segregated approach to synthesise the data; this involved analysing qualitative and quantitative data separately before integrating the results into a narrative summary (38, 39). The summary was scrutinised by the research team for accuracy.

## Results

### Study selection and characteristics

**FIGURE 1:**
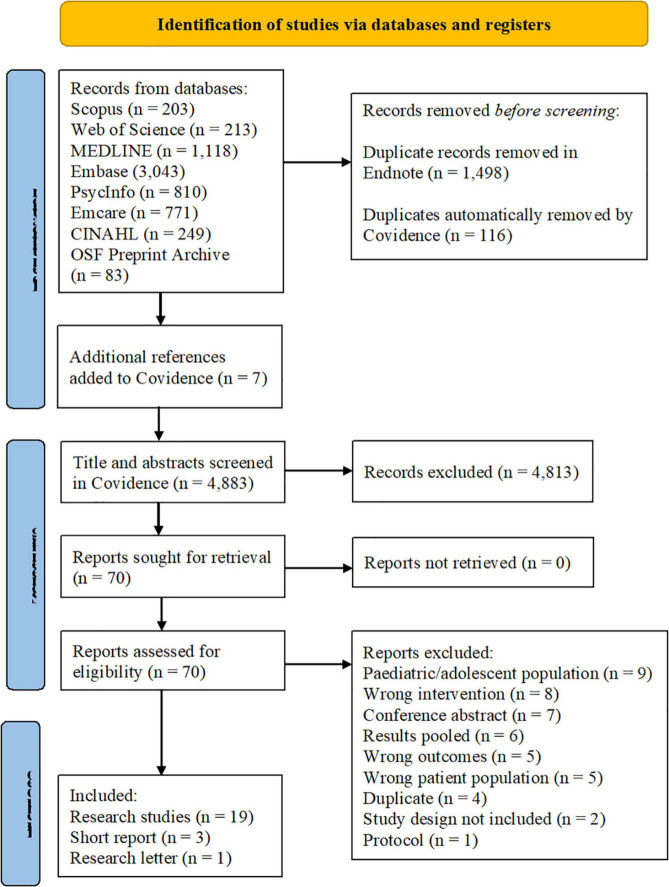
Screening process depicted in the PRISMA 2020 flow diagram (31).

We screened 4,883 titles and abstracts and assessed 70 full texts for eligibility. Twenty-three papers (40–62) met the eligibility criteria and were included in the review (see Figure 1).

The characteristics of studies included in this systematic review are presented in Table 2.

**TABLE 2:** Study characteristics.

References	Country	Condition	Study design	Sampling approach	Recruitment method	Primary outcome	Duration of follow up
Alinia et al. (40)	United States	Psoriasis	RCT	Convenience, purposive	Outpatient clinic	Treatment adherence*	12 months
Armstrong et al. (41)	United States	AD	RCT	Convenience	Outpatient clinic	Disease severity	3 months
Balato et al. (42)	Italy	Psoriasis	Randomised pilot trial	Convenience	Outpatient clinic	Treatment adherence*	3 months
Bundy et al. (43)	United Kingdom	Psoriasis	RCT	Voluntary	Advertisement	Anxiety and depression	6 months
Domogalla et al. (44)	Germany	Psoriasis	RCT	Convenience	Outpatient clinic	Anxiety and depression	60 weeks
Erdil et al. (45)	Turkey	AD (hand)	RCT	Convenience	Outpatient clinic	Unclear	2 months
Hawkins et al. (46)	United States	Psoriasis	RCT	Convenience	Outpatient clinic	Knowledge	Immediately post intervention
Heckman et al. (47)	United States	AD, psoriasis, chronic itch	Cohort study	Unclear	Market research company	Itch-related QoL	1 month
Hedman-Lagerlöf et al. (48)	Sweden	AD	RCT	Voluntary	Online application	Disease severity	12 months
Iliffe and Thompson (49)	United Kingdom	Alopecia	Qualitative	Voluntary, purposive	Social media	Patient experiences	No follow up
Joergensen et al. (50)	Denmark	AD	RCT	Voluntary	Social media	Disease severity, QoL	1 month
Koulil et al. (51)	Netherlands	Psoriasis**	Case report	Purposive	Unclear	Unclear	6 months
Lee et al. (52)	United States	Trichotillomania	RCT	Convenience, voluntary	University campus, mental health providers, online advertisement	Symptom severity, QoL	3 months
Manne et al. (53)	United States	Melanoma	RCT	Convenience	Cancer registry, dermatology clinics, medical centre	Skin self-examinations, sun protection behaviours	11 months
Marasca et al. (54)	Italy	Acne, alopecia, HS, lichen-plan-pilaris, psoriasis	Quasi-experimental study	Convenience	Outpatient clinic	QoL*	1 month
Mollerup et al. (55)	Denmark	AD (hand)	RCT	Convenience, voluntary	Outpatient clinic	Disease severity	6 months
Schuster et al. (57)	Germany	Psoriasis	Analytical cross-sectional study	Convenience, voluntary	Psoriasis	Unclear	No follow up
Sherman et al. (58)	Australia	Visible skin conditions including acne, birthmark, eczema, psoriasis, other	Randomised pilot trial	Convenience, voluntary	University campus, outpatient clinics, social media (Facebook)	Self-compassion*	Immediately post intervention
Svendsen et al. (59)	Denmark	Psoriasis	RCT	Convenience, voluntary	Outpatient clinic, advertisement	Treatment adherence	26 weeks
Russell et al. (56)	Australia	Melanoma	RCT	Convenience	Cancer centre	Unclear	6 weeks
van Beugen et al. (60)	Netherlands	Psoriasis	RCT	Convenience	Outpatient clinic, advertisement	Impact on daily life	6 months
van Cranenburgh et al. (61)	Netherlands	Acne, HS, psoriasis, vitiligo***	Observational pilot study	Convenience	Outpatient clinic	Acceptability, feasibility	2 months
Zhao et al. (62)	China	Psoriasis	RCT	Convenience	Outpatient clinic	Visit adherence*	12 months

AD, atopic dermatitis; HS, hidradenitis suppurativa; RCT, randomised controlled trial; QoL, quality of life.

*Primary outcome not explicitly stated by authors.

**This study also included one person with rheumatoid arthritis, but data were not included.

***Dermatologists were also recruited but data were not included.

We identified experimental studies, including 15 RCTs (40, 41, 43–46, 48, 50, 52, 53, 55, 56, 59, 60, 62), two randomised pilot trials (42, 58), one quasi-experimental design (54), as well as four observational studies (47, 51, 57, 61) and one qualitative study (49). The majority of studies were conducted in western countries; 11 in European countries (42, 44, 48, 50, 51, 54, 55, 57, 59–61) and six in the United States (40, 41, 46, 47, 52, 53).

Various sampling approaches were employed. Eleven studies utilised convenience sampling (41, 42, 44–46, 53, 54, 56, 60–62), four studies relied on voluntary sample (43, 48, 50, 57), and one study sampled purposively (51). Six studies used a combination of two sampling approaches (40, 49, 52, 55, 58, 59). One study did not clearly state how participants were sampled (47).

Twenty papers stated an eligibility criteria for participants, however, two papers (49, 54) did not provide an explicit criteria and one paper noted that the eligibility criteria was reported elsewhere (53). Several studies indicated a diagnosis by a clinician as a requirement for inclusion (43, 50, 57). Other studies specified people with a ‘diagnosis’ as an inclusion criterion but failed to clarify whether this was a self- or clinician-diagnosis (40, 45–47, 51, 55, 56, 58–61). However, given that research participants were mostly recruited from outpatient dermatology clinics (40–42, 44–46, 54, 55, 61, 62) or using a combination of recruitment methods (52, 53, 58–60), it is reasonable to assume that most studies included people with an established dermatological condition.

Few studies utilised established diagnostic criteria for determining eligibility for inclusion. Two studies relied on criteria for atopic dermatitis; one study (41) used criteria by Hanifin and Rajka (63) and the other study (48) employed The United Kingdom Working Party’s Diagnostic Criteria for atopic dermatitis (64). One study (52) determined the eligibility of people with trichotillomania for inclusion using the Diagnostic Statistical Manual of Mental Disorders 5 (DSM-5) criteria (65).

A number of studies only included people with determined severity using the following:

• Psoriasis Area Severity Index (PASI) (66) score of 5–15 (42).
• Mild to moderate psoriasis (43, 59).
• PASI and body surface area scores of >10 (44).
• Mild to moderate psoriasis judged as body surface area score of ≤10 (62).
• At least moderate severity according to the Patient-Oriented Eczema Measure (POEM) (67), defined as scores ≥8 (48).

The majority of studies were intended for people with specific dermatological conditions, including:

• Psoriasis (40, 42–44, 46, 51, 57, 59, 60, 62).
• Atopic dermatitis (41, 45, 48, 50, 55).
• Melanoma (53, 56).
• Alopecia (49).

One study (52) included people with Trichotillomania. Four studies were not condition-specific and were open to people living with different dermatological conditions, including, but not limited to, acne, vitiligo, hidradenitis suppurativa, and lichen-plan-pilaris, plus visible differences such as birthmarks (47, 54, 58, 61).

One sample included a parent of a person with alopecia (49) and one study recruited dermatologists in addition to patients (61). Another study described the case of a person with rheumatoid arthritis (51). These data were not included in the paper.

Sample sizes ranged from 2 (51) to 441 (53) participants. There were 2,268 participants across the studies and 556 participants were lost to follow-up. The total sample included 933 males and 1,132 females, although two papers did not report gender (46, 61). An overview of the number of participants and dropouts, as well as the gender and mean age of participants, are presented in Supplementary material, section 4.

A wide range of outcomes were studied, and a variety of measurement tools were used. Some psychological outcomes were assessed with established measures. For example, nine studies (42–44, 48, 50, 54, 55, 57, 59) measured QoL using the Dermatology Life Quality Index (DLQI) (68). One study (48) also used The Brunnsviken Brief Quality of Life Scale (BBQ) (69), and another study (52) employed the Quality of Life Scale (70). Validated measures of disease severity were also used widely: for example, six studies used the PASI (66); three studies (41, 48, 50) utilised the POEM (67); and four studies (42, 43, 51, 60) collected these data with the Self-Administered Psoriasis Area and Severity Index (SAPASI) (71). Several studies used non-validated self-report measures that had been developed for the purpose of the research being undertaken. These measures comprised of Likert (44, 53, 58, 61), numeric rating (40, 42, 44, 47, 51, 55), and visual analogue (45, 46, 48, 55) scales, as well as multiple choice (42, 46, 53) and true or false questions (45, 53). Fourteen studies (41, 43, 44, 46–50, 52, 53, 55, 59–61) specified at least one primary outcome and five studies alluded to a primary outcome (40, 42, 45, 54, 58, 62). The primary outcome could not be inferred for four studies (45, 51, 56, 57). All outcome variables studied, and measurement tools used in each study, are presented in Supplementary material, section 5.

Eighteen papers (40–45, 47, 48, 50–56, 59, 60, 62) included baseline measures and follow up periods varied substantially. Three papers conducted follow up immediately post-intervention (46, 58), although one study adopted a cross-sectional design meaning there was no baseline data to compare against (57). Other studies conducted follow up assessments after – 4 (47, 50, 54), 6 (56), 8 (13, 24, 26, 40–43, 45, 48, 51–53, 55, 59–62), and 60 (44) weeks post-intervention. Twelve papers assessed key outcomes more than once at the following timepoints:

• 1, 3, 6 and 12 months (40).
• After the 6-week intervention and 12 months (43).
• 12, 24, 36 and 60 weeks (44).
• 4 and 8 weeks (45).
• 3, 6 and 12 months (48).
• 9 weeks and 6 months (51).
• After fifth sessions, immediately post intervention, and 12 weeks following treatment (52).
• 8, 24 and 48 weeks (53).
• 2 and 4 weeks (54).
• 4, 8, and 26 weeks (59).
• 6 and 12 months (60).
• 2, 8, 16, 28, 48, and 52 weeks (62).

Seventeen studies included a comparator (40–46, 48, 50–53, 55, 56, 58–60, 62), mostly standard medical care (51, 56, 60), including drug treatments (40, 59), physical examinations (53), and written information about the condition of interest and treatment (41, 48, 55). Other control conditions included:

• A waitlist control group (43, 52).
• Use of electronic treatment dispensary caps (59).
• In-person follow-up visits (44).
• A standard writing activity (58).
• No intervention (45, 46).
• A matched control group (42).
• Daivobet^®^ (treatment) plus a mobile app without proactive communication with a doctor (62).

One study included two control groups; use of memory buttons only and no intervention (50).

### Methodological quality

Scores for methodological quality are presented in Supplementary material Table 3, section 6. Total quality scores ranged from two to 10, indicating that no paper met every criterion for their study design.

#### Levels of evidence

As for levels of evidence for effectiveness, papers were ranked to levels 1 (*n* = 16), 2 (*n* = 2), 3 (*n* = 1), and 4 (*n* = 2). Rankings ranged from level 1c (high quality) to 4d (very low quality). The two studies involving established qualitative methodology were both ranked to level 3 for meaningfulness (49, 55). Levels of evidence of effectiveness and meaningfulness are presented in Supplementary material Table 4, section 7.

#### Risk of bias

Seven papers (40, 41, 44, 47, 48, 59, 62) reported potential conflicts of interest and fourteen papers (40, 42, 43, 45, 49–51, 53–58, 60, 61) declared none. Two papers provided no information on this (46, 52).

Six studies were funded by pharmaceutical companies (40, 44, 47, 50, 61, 62), seven by public bodies (42, 43, 48, 49, 53, 55, 57), and three studies were funded by a combination of private and public organisations (51, 59, 60). Seven papers did not provide any funding information (41, 45, 46, 52, 54, 56, 58).

Blinding procedures were often poorly described or absent in reports of RCTs; in total, five papers explicitly described blinding procedures for participants (41, 48, 58) and treatment providers (42, 50), and only one paper covered blinding procedures for outcome assessors (42).

### Intervention characteristics

Intervention characteristics are presented according to the TIDieR checklist and guide (32) in Supplementary material, section 8. All interventions but one (49) were intended for individual use. Most interventions were delivered online via the internet (41, 43, 46–48, 51, 53, 56, 58, 60, 61), including the social media platform Facebook (49, 57). Five interventions utilised mobile technologies, including text messaging (42, 45) and mobile apps (62), or video conferencing software (52, 54). Five interventions comprised of two modes of delivery:

• Electronic medication canisters for monitoring psoriasis treatment, plus online reporting of disease status (40), or treatment information and reminders sent via a mobile app (59).
• Memory buttons and a mobile app for monitoring eczema treatment (50).
• Face-to-face education with an app for monitoring psoriasis (44).
• Face-to-face counselling and a website providing education, self-monitoring, and asynchronous communication for people with hand eczema (55).

Most interventions did not require a provider due to the focus on patient self-management. However, where involved, intervention providers included psychologists (48, 60), advanced graduate students supervised by a licensed psychologist (52), dermatology specialists (44), and nurses (55). The digital components of two interventions were not led by a provider (55, 60) and two papers did not describe the provider (51, 54). Only three papers gave sufficient detail about of the background, expertise, and suitability of the people responsible for intervention delivery (48, 52, 60).

Most interventions provided educational content on dermatological conditions and their management (41–46, 48, 51, 53, 55, 62) or:

• Psychological or social factors and coping (43, 48, 60, 61).
• Biological, psychological and social factors related to itch (47).
• Psychological factors related to trichotillomania and techniques for changing related cognitions and habits (52).
• Mindfulness (56).

Other features of digital interventions included:

• Text or email reminders prompting treatment (42) (45, 62) or intervention use (47, 55, 56).
• General assignments (43) and activities, for example, meditation (56) and writing a self-compassionate letter to oneself (58).
• Contact with intervention providers (44, 48, 51, 52, 55, 62) or patients (49, 55, 57).

Some interventions offered tailored content, including:

• Modules, assignments (55) and feedback, and goal setting (51, 60).
• Tracking physical (40, 44, 53, 55) and psychological (44) symptoms or treatment activity (50, 59).
• Allowing users a choice of modules to complete (61) and respecting personal treatment preferences (50).
• Individual counselling (55).
• Encouragement to verbalise reasons for performing sun protection behaviours and developing action plans (53).

Whilst intervention development was not the focus of this systematic review, we noted any descriptions of the theoretical foundations on which digital interventions were developed. Seven interventions endorsed established, evidence-based therapeutic approaches, including:

• Cognitive Behavioural Therapy (CBT) for psoriasis (43, 51, 60) or eczema (48).
• Acceptance and Commitment Therapy (ACT) Enhanced Behavior Therapy for trichotillomania (52).
• Self-compassion and written emotional disclosure (58).
• A mindfulness-based programme for melanoma (56).
• Habit reversal (51, 52).

Five of these digital interventions were based on existing protocols for face-to-face interventions (43, 48, 51, 52, 60). The authors of the written disclosure intervention (58) had adapted it from an existing intervention for breast cancer survivors. The web-based mindfulness programme (56) was built on a systematic review and the findings of a survey examining knowledge, attitudes and practices of meditation in people with melanoma.

In addition, parts of a web-based intervention (47) were based on the Biopsychosocial Model of chronic itch (72) and offered ‘cognitive-behavioural strategies’ for coping. One paper referenced using the Preventative Health Model (73) as a conceptual framework on which potential mechanisms of intervention effect could be based (53).

Other digital interventions were developed from:

• Expert medical knowledge of atopic dermatitis and its management (41).
• An existing educational intervention for psoriasis (44).
• A model of a German Tertiary Individual Prevention (TIP) clinical programme (55).
• ‘Previous research conducted by the research team’, including prototype testing of the electronic foam dispensers (SmarTop™) and smartphone app (MyPso SmarTop™) (59).
• An existing dermatology-specific measure of QoL (61) called Skindex-29 (74).

Three studies utilised existing digital technologies as part of their intervention, these included:

• Medication Event Monitoring System (MEMS^®^) caps (40).
• Memory buttons and a mobile app (50).
• A commercially available smartphone app (62).

The details of the development of some digital interventions were limited or absent from papers. For example, one text-based intervention delivered generic informational and motivational text messages to people with psoriasis, which were based on frequently asked questions and general recommendations for managing psoriasis, but the authors of the paper (42) did not give detail, including whether the motivational messages were underpinned by an existing theory or model of motivation. Another (50) drew links between their combined digital intervention and the Health Belief Model (75) in the discussion section of the paper, but did not expand on this anywhere in the methods section. One study developed an educational video on psoriasis onto an existing educational website for people with dermatological conditions, but no description of the development process was provided (46). The protocol for one intervention offering individual psychological video consultations was also not described (54).

### Results of intervention effectiveness

There were small bodies of evidence supporting the effectiveness of digital interventions for improving some ‘psycho-educational’ outcomes, particularly knowledge (41, 45, 46), mood (47, 51, 58) and the therapeutic relationship (42, 51, 52) to name a few.

The outcome variables studied and the associated findings for each study are presented in Supplementary material, section 9. We also recorded results relating to intervention usage, which are reported in Supplementary material, section 10.

#### Knowledge

The three studies (41, 45, 46) that assessed knowledge all reported significant improvements. One study found a significant improvement (*p* = 0.007) in the average knowledge scores between intervention (11/14) and control (9/14) groups immediately post-intervention (46).

Similarly, another study (41) showed significant improvement in knowledge in people who watched an educational video versus those who were given a pamphlet on atopic dermatitis at 12 weeks (3.05 vs. 1.85, *p* = 0.011).

One study (45) reported significant improvements in the knowledge level of people who did (14.8 ± 3.4) and did not (14.6 ± 3.9) receive a text-based intervention from baseline to 4-week follow up (*p* < 0.001 for both groups), although there was no significant difference in the change in knowledge levels between the two groups (*p* = 0.23).

#### Mood

All three studies (47, 51, 58) measuring affect detected positive results. One study (47) observed significant improvements in mean scores on the emotion subscale of ItchyQoL in people with atopic dermatitis, psoriasis and chronic itch through an educational website called Interactive Toolbox of Comprehensive Health Resources to Enhance Living with Itch (ITCH RELIEF) from baseline to 1 month (33.4 vs. 31.5, *p* < 0.01). A case report of an individual with psoriasis who received Internet-based CBT (ICBT) reported an improvement of at least 30% in negative mood from baseline to post-intervention, and at 6-month follow up (51). Similarly, individuals living with visible skin conditions demonstrated a significant improvement in mean scores for negative (baseline: 24.06 ± 7.90 vs. follow up: 22.21 ± 8.20, *p* = 0.028), but not positive affect, immediately after taking part in an online self-compassion writing activity, compared to those who participated in a standard online writing activity (58).

#### Therapeutic relationship

Four studies (42, 51, 52, 60) addressed the therapeutic relationship between patients and practitioners. Three of these studies indicated that different types of digital interventions can at least maintain (52), if not improve (42, 51), good working relationships between people with skin conditions and practitioners. One study (52) found mean scores for agreement on tasks and goals and the emotional ‘bond’ between participants and practitioners before and after treatment were higher than original scores, but no *p*-value was stated. The second study (51) reported improvements in mean scores pre and post ICBT intervention for agreement on treatment tasks (4.25 vs. 4.75) and goals (4.5 vs. 4.75) yet no *p*-value was reported. The third study (42), however, did not report the statistics or *p*-values used to test this variable. The final study found that positive perceptions of the therapeutic alliance at the outset of ICBT treatment predicted significant improvements in physical (*p* = 0.02) and psychological (*p* < 0.001) outcomes (60).

#### Anxiety

Five studies explored anxiety and reported mixed results. One study (43) observed a significant reduction in mean anxiety scores from baseline (7.6 ± 3.6) to 6-month follow up (6.1 ± 3.5) in people with psoriasis compared to controls (*p* < 0.05), whereas two studies reported no group differences in general anxiety scores (*p* = 0.24) (48) or anxiety as a composite component of psychological functioning (*p* ≥ 0.20) (60). One study (51) found improvement of at least 30% in anxiety scores post ICBT treatment but were not maintained long-term follow up, although no significance value was reported. A significant improvement in anxiety scores were found in another study (44) after 12 (*p* = 0.02) and 24 weeks (*p* = 0.01) but not after 36 (*p* = 0.08) or 60 (*p* = 0.06) weeks.

#### Depression

Similarly, the evidence for depression varied. Significant between-group differences (reductions) in depressive symptoms were reported in people with psoriasis (*p* < 0.05) (44) and atopic dermatitis (*p* = 0.008) (48) from baseline to 12 weeks post treatment.

Another study (43) found that the proportion of people with psoriasis who were considered to be clinically depressed fell from 15.5% to 2.3% following the eTIPs intervention, yet the difference in depression scores between the intervention and control groups was not statistically significant for either the complete cases (*p* = 0.088) or following multiple imputation analysis for missing data (*p* = 0.34). In addition, no significant differences in depression were found between participants who received ICBT and those who did not from baseline to post treatment or 6-month follow up (*p* ≥ 0.20) (60). One individual with psoriasis showed an improvement in depression of at least 30% from baseline to post treatment assessment, but no significance value was stated (51).

#### Treatment adherence and compliance

Eight studies measured adherence to treatment (40, 42, 45, 46, 51, 59, 60, 62). The first study (40) found post-treatment rates of adherence were significantly higher for participants in the internet survey group compared to the control group from the first to the tenth month (*p* = 0.03), after which adherence rates declined for both groups. The second study (42) found that treatment adherence increased in the experimental group only from 3.86 days per week at enrolment to 6.46 days per week following the text message intervention (*p* < 0.01). Another study (46) reported that participants were not more likely to report using their medication as prescribed after accessing an educational psoriasis website (no significance value given). The next study (59) found, according to the main analysis of chip adherence data, more patients in the intervention group were adherent than patients in the non-intervention group (65% vs. 38%, *p* = 0.004). This study also claimed that patient reported adherence to cutaneous foam was higher in the intervention group (14%) compared to the control (8%) after 1 month, but the difference was not statistically significant (*p* = 0.069) (59). One study (62) found that 13/41 (31.7%) participants who completed a follow up survey at week 12 reported using Daivobet^®^ sometimes or never in the previous 4 weeks. Three studies (45, 51, 60) referred to treatment compliance. One study (45) found no statistically significant difference between the number of participants in the text-based intervention and control groups who forgot to use their medication (52.9% vs. 64.7%, *p* = 0.33). No significant change in the maximal treatment compliance score was observed in an individual with psoriasis from pre to post intervention or follow up (51). Nor did treatment compliance differ significantly between participants who received ICBT or standard care at pre, post or follow up assessment (*p* ≥ 0.25) (60).

#### Skin protection behaviours

As for skin protection behaviours, one study detected significant improvements in moisturiser use from baseline to week 4 (*p* < 0.001) and 8 (*p* = 0.020), in the text-based intervention group (45), although the use of moisturiser was significantly higher in the intervention versus control group at week 4 only (*p* = 0.008). In another study (55) people with hand eczema who received a combined face-to-face counselling and website intervention reported a significant change in the mean scores for performing habits relating to their condition (e.g., using topical steroids and consulting General Practitioner) compared to participants who did not have access to the website (7.9 ± 2.4 vs. 6.6 ± 3.2, *p* = 0.024). This was the case for people with melanoma who participated in the mySmartSkin intervention, who reported performing significantly more sun protection behaviours on average at 24 weeks (i.e., sunscreen use, wearing hats and long sleeves, and seeking shade) compared to controls (3.54 ± 0.74 vs. 3.37 ± 0.84, *p* = 0.031) (53). Greater knowledge of melanoma and increased self-efficacy both partially mediated the relationship between intervention use and performing sun protection behaviours (53). Two studies recorded scratching behaviour using different measures; one study reported significant within-group reductions from baseline to 1-month follow up in mean scores for scratch intensity (12.3 vs. 11.6, *p* < 0.05) and impact (19.8 vs. 17.9, *p* < 0.001), and sleep-related itch and scratch (37.4 vs. 133.3, *p* < 0.001) (47). The other study (51) reported a reduction in scratching behaviour in a person with psoriasis, but the authors did not specify whether the change reached the threshold for statistical significance.

#### Physical outcomes

A similar picture was observed for physical outcomes. There was clear evidence for improving disease severity in the short term (1–3 months). One study (40) detected significant improvements in PASI, but not Investigator Global Assessment, scores between the intervention and control group after 1 (1.61 vs. −0.12, *p* = 0.003), 3 (2.50 vs. 0.79, *p* = 0.025), and 12 (3.32 vs. 0.34, *p* = 0.038) months. Another study (59) found a significant improvement in psoriasis severity in the intervention group from baseline to week 4. One study found no significant difference between SAPASI scores of participants who tested the eTIPs intervention and those who did not for either the complete cases (*p* = 0.67) data or multiple imputation analysis for missing data (*p* = 0.92) (43). Significant mean reductions in hand eczema severity scores were seen after 8 weeks in participants who received a text message intervention compared to the control group (70.2% ± 35.2 vs. 38.9% ± 67.7, *p* = 0.017) (45). At 12 weeks, greater improvements in the severity of atopic dermatitis were observed in people who viewed an educational video online compared to those who read an educational pamphlet (3.30 vs. 1.03, *p* = 0.0043) (41). Following receipt of a text-based intervention, people with psoriasis also reported significantly reduced (*p* < 0.05) disease severity [PASI, SAPASI, Physician Global Assessment (PGA), and body surface area] at 12 weeks compared to controls (42). Lastly, significantly larger reductions (*p* < 0.005) in scores of objective measures of disease severity [Eczema Areas Severity Index (EASI) and SCORing Atopic Dermatitis (SCORAD)] were observed in people who received electronic memory buttons plus an app, compared to the two control groups, as was a significant decrease (*p* < 0.05) in subjective POEM scores at the second consultation approximately 1 month after participants began using the intervention (50).

Evidence for effectiveness beyond 6 months was mixed. One study (40) observed a significant improvement in PASI scores in the intervention group at 12-month follow up compared to the control group (3.32 vs. 0.34, *p* = 0.038) until alcohol use and smoking status were included in the analysis as covariates. Similarly, people with eczema who trialled ICBT showed a significantly greater reduction (*p* < 0.001) in average weekly symptoms measured by POEM at 12-month follow up compared to the control group (48). Another study showed that clinician-assessed disease severity worsened slightly between baseline and 6-month follow up but no significance value was reported (51). One study did not detect a significant difference (*p* = 0.16) in median hand eczema severity index (HECSI) scores of website and non-website users (55).

Improvements in psoriasis severity were noted in the longer term in two studies; the first study (44) reported significant reductions (*p* < 0.001) in PASI scores in all patients from baseline to follow up at week 60, but no group effect was found. The second study (59) found that the greater improvement in psoriasis severity, measured by the lattice system physicians global assessment (LS-PGA), that was observed in the intervention group in the short term, no longer reached the threshold for statistical significance at week 8 or 26.

Reductions in itch were also seen at 4 weeks (*p* < 0.001) (47), after 6 months (*p* = 0.052) (55) and 12 months in people with atopic dermatitis (*p* = 0.01) (48). One study (44) found itch significantly reduced in all participants with psoriasis after 60 weeks, although the difference between the groups was not statistically significant. One study did not control for use of itch medication (47).

#### Quality of life

As for QoL, two studies (47, 54) reported significant within-group differences from baseline to 4-week follow up. The first study was specific to itch-related QoL (78.9, 95%, confidence interval [CI] = 75.9–81.9) to follow up (75.4, CI = 72.4–78.5, *p* = 0.007) (47). The second study employed the DLQI (4.4 ± 3.9 vs. 1.6 ± 2.5, *p* < 0.05).

Three studies detected significant between-group differences in QoL favouring the intervention group, from baseline to week 6 (*p* = 0.042) (43), week 12 (*p* < 0.05) (42), and after 6 months (*p* = 0.014) (55). One study (48) found a significant between-group difference in QoL favouring the ICBT intervention group with the BBQ (*p* = 0.001) (69), but not the DLQI (*p* = 0.07) (68).

Two studies (44, 52) reported improvements in QoL that did not reach statistical significance. Another study noted a reduction in DLQI scores in the intervention group compared to controls at weeks 4 and 8, which relapsed at week 26, yet none of these group differences reached the threshold for statistical significance (59).

#### Other psychological outcomes

Various psychological concepts were measured in one study only. The high level of heterogeneity in the outcome variables studied meant evidence was often not sufficient to make general claims about specific outcome variables. Statistically significant reductions were found for the following outcomes:

• Perceived helplessness in one individual living with psoriasis (significance value not reported) (51).
• Fear of cancer recurrence in people who received an online mindfulness-based programme, compared to controls (mean difference: −2.55; 95% CI = −4.43 to −0.67; *p* = 0.008), but only few of these scores fell below the clinical cut-off (≥13) (56).
• Perceived stress (*B* = 5.09; 95% CI = 1.96–8.21; *z* = 3.19; *p* = 0.001) and sleep problems (*B* = 3.38; 95% CI = 1.28–5.48; *z* = 3.15; *p* = 0.002) in people who received ICBT versus the control group (48).
• Trichotillomania severity from pre to post ACT Enhanced Behavior Therapy via telepsychology [slope estimate = −6.13, SE = 1.30, *t*(58.48) = −4.72, *p* < 0.001] (52).

One study observed a statistically significant improvement in mean self-compassion scores (*p* = 0.006) in people with visible skin conditions following an online self-compassion writing activity (3.33 ± 0.60), compared to those who participated in a standard online writing activity (2.84 ± 0.62) (58).

A number of these papers reported trends towards improvement but were not statistically significant. These outcomes included:

• Self-efficacy for managing eczema in website users versus non-website users (*p* = 0.093) (55).
• Rumination in people with melanoma following an online mindfulness programme compared to controls (mean difference: −2.76; 95% CI = −6.67 to 1.17; *p* = 0.169) (56).
• Impairment in daily activities following an educational session via a psoriasis management smartphone app, and participants in the control group (*p* = 0.63) (44).
• Psychological well-being of people with skin conditions following psychological video consultations (baseline: 68.5 ± 15; week 4: 77.1 ± 16; no *p*-value reported) (54).
• Psychological flexibility scores post ACT Enhanced Behavior Therapy via telepsychology [*F*(1,18) = 3.790, *p* = 0.068, ω^2^ = 0.064] (52).

There were several psychological outcomes for which no significant between-group differences were reported:

• Perceived stress (*p* = 0.719) or worry (*p* = 0.814) in people with melanoma who attempted mindfulness and those in the control group (56).
• Anxiety, depression and negative mood (all *p* > 0.20), or psychological functioning overall (*p* = 0.32), in people with chronic skin conditions following ICBT and those in the control group (60).
• The rates of hospital visits in people with psoriasis who received a smartphone app with or without prompted communication from doctors (5.2–15.7% vs. 7.5–17.0%, *p* > 0.05), although older age (50 to 60 years: *P* = 0.02) and greater body surface area (scores 7 to 10: *p* = 0.02), were associated with more hospital visits (62).

One case study tracked changes in psychological and social outcomes overtime in someone with psoriasis who received ICBT and found that high and low levels of social support and stigma (respectively), and maximal impact of psoriasis on daily life, remained unchanged from baseline through to follow up (51).

Individual studies also produced mixed findings for specific outcomes. For example, a study of ACT Enhanced Behavior Therapy delivered via video conferencing showed decreases in shame scores that did not differ significantly when comparing the intervention and control groups. However, when the groups were entered into a combined analysis, a significant change in shame scores was observed from post-treatment to follow up only (*p* = 0.002) (52).

Another study used a composite measure of impact on daily life, which was comprised of physical and psychological functioning and role limitations due to physical and emotional health problems, as a measure of impact on daily activities (60). After 6 months, significant improvements were observed for role limitations due to emotional and physical health problems (both *p* = 0.04) in individuals receiving ICBT, compared to other participants who received care as usual. The improvement in role limitations due to emotional problems was further enhanced at follow up (*p* = 0.047). However, no significant difference (*p* ≥ 0.17) in role limitations was found between the groups when baseline values of the dependent variable were included in a secondary analysis.

One study reported that the significant between-group difference in PASI scores favouring the intervention group (*p* = 0.038) at 12 months no longer reached statistical significance when alcohol consumption and smoking status were controlled (*p* = 0.07) (40).

Other independent studies included measures of psychological outcomes but were limited for different reasons. Firstly, one study found that higher levels of Facebook envy were associated with lower levels of life satisfaction (standardised coefficient [β] = −0.38, CI = −0.58 to −0.16) and happiness (β = −0.36, CI = −0.57 to −0.14) in people with psoriasis. This study was cross-sectional and thus Facebook envy and potentially relevant factors could only be measured at one timepoint.

One study measured the average number of minutes that people with melanoma reported meditating per week across a 6-week online mindfulness programme (56). This varied greatly from 64 min in week 2 to 129 min in week 5, but the authors did not test for statistically meaningful differences in the average meditation times at different timepoints.

Lastly, two papers reported measuring psychological outcomes, specifically participants’ beliefs about psoriasis (43) and self-efficacy to interact with clinicians (47), but the results for these outcomes were not reported.

### User views and experiences

In total, 13 studies explored people’s views and experiences of digital psychological interventions (41, 42, 46, 48, 49, 51–53, 55, 56, 59–61). Of these studies, only one adopted a purely qualitative design, (49) and others:

• Included a qualitative component, but only referred to the study as a mixed-methods study in the discussion section (55).
• Described a qualitative content analysis, but did not label the analysis as such (56).
• Did not describe how qualitative data were analysed (46).

The synthesis is reported in relation to acceptability and feasibility, satisfaction, positive feedback, perceived benefits, and barriers to digital intervention use.

In terms of the acceptability and feasibility of digital psychological interventions, two studies (56, 61) explicitly aimed to explore intervention acceptability and feasibility. The first study (56) found that an online mindfulness intervention was acceptable to people with melanoma, as 23/32 (72%) respondents deemed the intervention to be helpful. Furthermore, 70% of participants completed the end-of-study questionnaire and most participants noted that the intervention was simple to use, demonstrating intervention feasibility (56). The second study (61) reported that people with visible skin conditions considered an online educational website appealing and convenient, but overall acceptability was lower than expected because users did not think the website content was relevant to them. It was concluded that this intervention was not feasible overall because users either somewhat or totally agreed that their daily activities prevented regular use (61).

Seven studies measured how satisfied people living with psoriasis (42, 46, 51, 60), atopic dermatitis (41, 48), and trichotillomania (52) were with the interventions they received. These studies indicate high levels of user satisfaction, and that users would recommend, continue using (42, 46), and might prefer online interventions in future (51, 60).

Six studies (41, 42, 46, 51, 52, 60) captured positive feedback from users, which lends further support to the acceptability and feasibility of digital psychological interventions. Users remarked on the user-friendliness (51, 60), appeal (41), convenience (51, 52), and usefulness (42) of digital psychological interventions, particularly for understanding dermatological conditions (46).

A range of perceived benefits of using digital psychological interventions were reported by users across five studies (49, 51, 53, 55, 56). People reported that interventions of this kind improved their knowledge of their condition and sense of personal control (53, 55).

In addition, these interventions were seen to facilitate positive psychological well-being by helping individuals to accept (56) and regulate their feelings (e.g., helplessness, depression) (49, 51, 53) and behaviour (e.g., itch), and identify coping strategies (51). The benefits of online peer support included facilitating emotional expression, self-confidence and acceptance, and exchanging knowledge, experiences and tips for coping and management (49).

Four studies identified barriers to digital intervention use. These barriers included technical problems (e.g., difficulty accessing and navigating the intervention) and individual factors (e.g., personal priorities, preferences and schedules, physical symptoms, geographical location, and a lack of time) (53, 55, 56, 61). One study (55) found that certain features, specifically digital reminders and interactive activities, facilitated the use of digital interventions.

### Integration of qualitative and quantitative results

We identified some overlap between qualitative and quantitative data for some outcomes. Firstly, knowledge of skin conditions and their management. Quantitative data revealed significant improvements in participants’ knowledge following the use of digital psychological interventions, including an online educational video on eczema (41), and a text message intervention (45) and an online educational website (46) for psoriasis. Two studies (46, 53) involving patient evaluations also found participants felt more informed about their conditions and how to manage them following intervention use, and a group intervention enabled members to share knowledge and learn from each other (49).

Secondly, we identified some parallels between the quantitative and qualitative data relating to emotions. The former indicated that use of digital interventions, including ICBT (51), online self-compassion writing (58), and an educational website (47) improved negative mood in particular. One qualitative study (49) similarly found that an online support group enabled people to express how they felt about alopecia. In addition, feedback from people with melanoma suggested that they felt calmer, at peace and more at ease after taking part in online mindfulness (56).

Another outcome for which there was congruence was stress. One study (48) found significant reductions in perceived stress among the ICBT intervention group versus controls. This was supported by one study (56) in which eight reports from five participants suggested an online mindfulness intervention helped individuals to manage their stress.

We did not identify any contradictory evidence. Many of the outcome variables measured in quantitative studies were not addressed in the few qualitative studies that were included in this review.

## Discussion

As digital technology becomes further embedded in health care generally, this mixed methods systematic review offers valuable insight into the potential effectiveness of digital platforms and content for improving some psychological and physical outcomes in people with dermatological conditions, mainly psoriasis and eczema. There is some support for web-based digital interventions to improve people’s knowledge of their skin conditions and its management, and emotional functioning, particularly negative affect. Use of digital interventions also seemed to benefit aspects of disease severity in the short to medium term. These insights align with some of the findings of an earlier meta-analysis of effectiveness of psychological interventions for adults with skin conditions, which detected medium effect sizes for psychological outcomes and skin severity (76).

We identified several digital interventions that focused on treatment non-adherence, a significant problem within dermatology (77). However, most of these interventions did not lead to significant improvements in treatment adherence and therefore a new approach is needed.

Some digital interventions showed improvement in QoL and offers some confidence that digital interventions requiring active involvement from a provider (e.g., ICBT) are at least as good as those delivered in person in terms of facilitating rapport between the people receiving and delivering the intervention. This is a useful finding given that previous research with people with psoriasis (78) and hidradenitis suppurativa (79) have indicated that other forms of digital interventions, including remote consultations via video, and telephone consultations especially, are not conducive to discussing the broader psychological impact of skin conditions or building rapport between patients and clinicians.

Overall, considerable heterogeneity in study designs, measures and outcomes meant there was a lack of sufficient and consistent evidence for many psychological outcomes preventing us making definitive conclusions about intervention effectiveness. The level of diversity within this systematic review mirrors that found in a previous systematic review of psychological therapies in psoriasis management (80). Several papers indicated any suggested improvements did not reach the threshold for statistical significance; it is plausible that some of the studies reviewed were not sufficiently powered, as also suggested by another previous systematic review and meta-analysis of psychological and education interventions for atopic dermatitis specifically (81).

As for people’s views and experiences, we found poor reporting of qualitative methodology in some studies that sought patient evaluations. Some, mostly web-based interventions, may be acceptable to people living with different dermatological conditions but personal factors could also present as barriers to intervention use. The main benefits of digital interventions included improved emotional control (82) and confidence to socially interact (83), which echo similar findings of previous research (82, 83). A better understanding of dermatological conditions and approaches to management were also a key benefit of digital interventions. Importantly, some of these key qualitative findings lend support to the positive quantitative results showing improved knowledge and emotional functioning. Furthermore, the qualitative and quantitative insights on user knowledge that we have identified arguably builds on previous research, which was unable to determine the efficacy of educational and psychological approaches for adults with atopic dermatitis (81). The present review gives us some confidence that digital interventions including educational material are likely to be of some benefit to people with dermatological conditions, the next step is to find out what benefit and for whom.

### Strengths and limitations

To our knowledge, this is the first mixed methods systematic review investigating *digitally delivered* interventions supporting the psychological health of people with dermatological conditions. The TIDieR checklist and guide (32) provided a comprehensive framework for charting key characteristics of the digital interventions clearly, and identifying gaps in reporting. This review was conducted by a multi-disciplinary team of health and clinical psychologists and a general practitioner, most of whom specialize in dermatology research and practice. It was supported by experts from a JBI Centre of Excellence and followed JBI methodology; JBI is renowned for the conduct of highly rigorous evidence syntheses to promote and implement evidenced-based decisions to improve health and healthcare globally (84). The use of JBI critical appraisal tools allowed for a detailed and nuanced assessment of different study designs. In addition, it has been noted by experts in JBI methodology that the step of corroborating and refuting findings is often lacking or missing entirely from mixed methods systematic reviews (38). We adopted a convergent segregated approach to data synthesis and as a result were able to triangulate some of the key findings relating to cognitions and emotions specifically, further strengthening the present review.

However, our decision to review all eligible studies regardless of quality meant three short reports (42, 47, 54) and one research letter (46) were included, arguably weakening the overall quality of this review. We also opted to include a paper specific to trichotillomania; a complex psychiatric disorder (85). Whilst this inclusion constitutes as a deviation from the protocol, people with trichotillomania often present to dermatology staff, psychiatrists and psychologists (86), reiterating the complex interplay between dermatological and psychological factors. Thus, we argue that the contents of this paper on trichotillomania are likely to be of relevance to the dermatology community, justifying its inclusion in this systematic review. Furthermore, we identified several papers at the full text screening stage which were of some relevance to this review, but these were excluded on the basis that they involved people as young as 12 (15, 87–90) and 16 (91, 92) years old and pooled the results (93–96), preventing us from extrapolating the results specific to our population of interest. It is possible that we missed information related to the review questions by excluding these papers. Lastly, two of the papers included in this review were authored by CB (43) and AT (49), potentially introducing bias. However, we attempted to counter this bias by ensuring that neither author was responsible for reviewing their respective papers at any point in the review process.

### Future research

Further work to design and test digital psychological interventions is needed, as is qualitative research, to ensure future interventions are feasible, appropriate, meaningful and effective (84) for people with a broad range of common and rare dermatological conditions (97). We have shown that existing research largely focuses on specific dermatological conditions, mainly psoriasis followed by eczema. Researchers should aim to develop digital interventions targeting other dermatological conditions, such as hidradenitis suppurativa and acne, which carry a substantial psychological burden (8), as well as digital interventions that tackle psychological impacts that are common across dermatological conditions. The TIDieR checklist and guide (32) is likely to be a useful tool for intervention developers to consider when planning, developing, and particularly when reporting, complex digital interventions.

This review highlights that many existing studies lack quality, despite the level of evidence they were assigned to. In the context of RCTs, for example, these studies were ranked to level 1, the highest level of evidence for effectiveness, but most were missing detailed information about standard trial procedures, such as blinding. This criticism aligns with earlier research calling for a higher quality and better reporting of RCTs (76). Underreporting of blinding procedures in RCTs of psychological interventions is not a new finding, but it is paramount that researchers explore all possible avenues for blinding, adequately report blinding attempts, and acknowledge potential pitfalls where blinding is not possible (98). Greater transparency in the reporting of these procedures could facilitate the development of more robust RCTs in the future, and support healthcare professionals and policy makers to make more informed, evidence-based decisions relating to the care of people with dermatological conditions.

Furthermore, it seems that larger samples might be required for future studies of digital interventions to determine whether their use can significantly improve psychological outcomes (e.g., self-efficacy, well-being, etc.) in people with dermatological conditions, and to establish the magnitude of the effect where one exists.

We also emphasise the need for more qualitative research to further explore intervention barriers and facilitators to using digital psychological interventions and outcomes that are meaningful to patients. Addressing these issues directly with people living with a range of dermatological conditions, as well as ways of overcoming barriers to use, could help to maximise the appropriateness, practicability, and usability of new digital psychological interventions for this population (28). The qualitative data offers some insight into psychological factors (e.g., personal control and acceptance) which might help to explain the mechanisms through which digital interventions work, as does qualitative and quantitative data on self-efficacy and knowledge. It is important to investigate these factors further to determine whether they are indeed mechanisms for change. However, qualitative methodologies were sometimes not acknowledged or described sufficiently by authors. Thus, more explicit and comprehensive reporting of qualitative methodologies is required.

### Practical implications

Several studies focused on treatment behaviours. Whilst treatment adherence and skin protection are important for managing dermatological conditions (77), other modifiable dietary and health behaviours, such as smoking, alcohol consumption, and poor sleep are associated with some, mostly inflammatory, dermatological conditions (99), and increased risk of cardiovascular disease (100–102). Digital interventions addressing a variety of health behaviours are, therefore, needed to support a holistic and effective approach to patient self-management.

While many studies in this review included an educational component, the provision of information alone is not always sufficient for eliciting behaviour change; other factors, including personal capabilities, opportunities and levels of motivation, are established drivers of behaviour (103). In the context of treatment adherence, for example, other psychological factors, such as illness and treatment beliefs and concerns, are known to influence behaviour (77). Dermatologists involved in developing digital interventions should address the psychological factors which underpin adherence to dermatological treatments (77), as well as target other related health behaviours.

Whilst intervention development in the usual way was not the focus of this systematic review, it was not always clear from the papers included if or how theoretical frameworks contributed to intervention development, or if the perspectives and needs of the target user were considered throughout this process. Digital behaviour change interventions, like face to face interventions, should be informed by theory in order to determine and test mechanisms for change (104). The Behaviour Change Wheel (BCW) is an example of an established and evidence-based framework for designing behaviour change interventions (103). At the heart of the BCW sits the COM-B Model, which encapsulates three key drivers of behaviour: Capability, Opportunity and Motivation (103). The BCW also specifies nine intervention types and seven policy categories that could aid the design and implementation of new interventions (103). Specialists in dermatology should adopt behavioural science principles, including recognised theories of behaviour change, such as the COM-B Model (103), and a person-based approach from the outset, to ensure digital interventions meet the needs and preferences of people living with dermatological conditions (104). We also advocate for interdisciplinary collaborations between experts in dermatology, technology, and particularly behaviour change, to facilitate better understanding, development and testing of future complex digital interventions (104).

## Conclusion

This mixed-methods systematic review shines light on a diverse range of existing digital psychological interventions for some dermatology conditions, as well as substantial heterogeneity and varying quality in the literature. A lack of sufficient and consistent evidence allowed for, at best, tentative conclusions on intervention effectiveness. Whilst digital interventions of this kind are, to some extent, acceptable to patients, there are barriers to their use, and these must be addressed to maximise future use. Collectively, existing evidence underscores the need for quality and interdisciplinary research to develop and test complex digital psychological interventions targeting a broader range of psychological factors, specifically health behaviours, with input from people living with dermatological conditions.

## Data availability statement

The original contributions presented in this study are included in the article/Supplementary material, further inquiries can be directed to the corresponding author.

## Author contributions

RH contributed to the conceptualisation, lead for methodology, material development, database searching, article screening, data extraction, curation, critical appraisal, data synthesis, writing – original draft and review, and editing, and project administration. MP contributed to the database searching, article screening, data extraction, critical appraisal, and writing – review and editing. CP contributed to the conceptualisation, methodology, consensus checks and discrepancy resolution for article screening, data extraction and critical appraisal, and writing – review and editing, and supervision. RP contributed to the conceptualisation, methodology, article screening, data extraction, critical appraisal, discrepancy resolution, consensus checking, and writing – review and editing. BJ contributed to the methodology, material development, article screening, data extraction, critical appraisal, consensus checking, and writing – review and editing. GW and OH contributed to the material development, article screening, data extraction, critical appraisal, consensus checking, and writing – review and editing. MR contributed to the consensus checking and discrepancy resolution for article screening, data extraction and critical appraisal, and writing – review and editing. AT contributed to the consensus checking and discrepancy resolution for article screening and data extraction, and writing – review and editing. CB contributed to the lead for conceptualisation and supervision, supported methodology and writing – review and editing. All authors contributed to the article and approved the submitted version.
