# Supplementary material for: A mixed methods systematic review of digital interventions to support the psychological health and well-being of people living with dermatological conditions
Source: Front Med (Lausanne). 2022 Nov 3;9:1024879. doi: 10.3389/fmed.2022.1024879 (PMC9669071; doi:10.3389/fmed.2022.1024879)
Supplement: Supplementary file 1 [file Data_Sheet_1.docx]

Supplementary Material

# Search strategy for MEDLINE (Ovid)

exp Skin Diseases/
exp Skin Neoplasms/
exp Dermatology/
((Skin or dermatolog* or cutaneous or visible) adj2 (condition* or disease* or disorder* or manifestation* or difference*)).ti,ab.
1 or 2 or 3 or 4
exp Mobile Applications/
exp Cell Phone/
exp Internet-Based Intervention/
exp Telemedicine/
((mobile* or cell* or smart* or text* or tablet) adj3 (phone* or device* or app or apps or application* or message* or comput*)).ti,ab.
(iPhone* or i-Phone* or iPad* or i-Pad* or email or e-mail).ti,ab.
((internet* or web* or online* or comput*) adj3 (device* or app or apps or application* or program* or intervention*)).ti,ab.
(ehealth or e-health or "electronic health" or mhealth or m-health or "mobile health" or etool* or e-tool* or e-diar* or elearning or e-education).ti,ab.
(digital adj3 (health or intervention* or technolo* or program* or device* or app or apps or application*)).ti,ab.
((remote or virtual or tele) adj3 (health* or intervention* or program* or consultation* or counsell* or therap*)).ti,ab.
("social media" or facebook or Instagram or twitter or whatsapp or tiktok or instant messag*).ti,ab.
6 or 7 or 8 or 9 or 10 or 11 or 12 or 13 or 14 or 15 or 16
exp "Quality of Life"/
exp Health Behavior/
exp Illness Behavior/
exp Social Behavior/
exp Self-Management/
exp Self Care/
exp Stress, Psychological/
(quality adj4 (health-related or life or adjusted Life Years)).ti,ab.
((cognitive or psycholog* or psychosocial or emotion* or mental* or behavio?r or social or daily) adj3 (adjustment* or function or support or health or well-being or wellbeing)).ti,ab.
(emotion* or mood or distress or motivation* or depression or anxi*).ti,ab.
((illness or treatment) adj3 belief*).mp.
((behaviour* or behavior*) adj3 (health or change or illness or coping)).ti,ab.
(self* adj2 (manage* or help or care or efficacy or esteem or compassion)).ti,ab.
(alcohol or smoking or smoking cessation or exercise or physical activity or diet or healthy eating or eating behavio?r or weight loss or weight maintenance or weight management or sleep or adherence or treatment adherence or meditat* or mindfulness or yoga).ti,ab.
(daily adj2 (living or function or activit*)).ti,ab.
(social adj2 (support or adjustment or connect* or interaction* or function or activit*)).ti,ab.
18 or 19 or 20 or 21 or 22 or 23 or 24 or 25 or 26 or 27 or 28 or 29 or 30 or 31 or 32 or 33
5 and 17 and 34
limit 35 to (english language and yr="2002 -Current")

# Screening tool

Section A

Author(s): ID:

Year: Country:

Reviewer: Date reviewed:

Instructions: Assess the article against the criteria below and tick one box for each statement. Please state your final decision in Section G and provide a brief explanation.

Section B

The study…

Is written in English ❑ Yes (continue screening) ❑ No (exclude)

Was written/published in or after 2002 ❑ Yes (continue screening) ❑ No (exclude)

Section C

The participants…

Are aged 18+ years ❑ Yes (continue screening) ❑ No (exclude)

Have a dermatological condition ❑ Yes (continue screening) ❑ No (exclude)

If yes, state name of condition(s):

Section D

A digital intervention for patients that includes at least one of the following functions:

❑ Yes (tick all functions below that apply and continue) ❑ No (exclude)

*Delivered by digital technology* ❑
*Can be accessed online or offline* ❑ *Patient-to-patient communication* ❑ *Patient-to-practitioner communication* ❑ *On-demand information services* ❑
*Personal health tracking* ❑ *Targeted patient communication* ❑

A digital health intervention(s) for symptom detection, diagnosis, examination or assessment ❑ Yes (exclude)

Intervention delivered via telephone only ❑ Yes (exclude)

Asynchronous telemedicine (e.g., Store and Forward) only ❑ Yes (exclude)

Section E

This study concerns the following outcomes (tick all that apply) …

Cognitive (e.g., beliefs about illness or treatment) ❑ Yes ❑ No

Emotional (e.g., depression, anxiety, stress) ❑ Yes ❑ No

Behavioural and behaviour change (e.g., diet and weight ❑ Yes ❑ No
management, physical activity or exercise, smoking, alcohol consumption,
sleep, medication adherence).

Other psychological outcomes (e.g., self-efficacy, self-compassion, ❑ Yes ❑ No
motivation, quality of life, and adjustment).

Other outcomes (e.g., intervention feasibility, acceptability or ❑ Yes ❑ No
usability, and user satisfaction or engagement) ***in addition to*** one or
more psychological outcome.

Physical outcome (e.g., pain severity, duration, skin coverage) ***in*** ❑ Yes ❑ No ***addition to*** one or more psychological outcome.

Usage data metrics (e.g., number of log ins, modules accessed, ❑ Yes ❑ No
time spent on/ using digital intervention) ***in addition to*** one or more
psychological outcome.

Physical outcome only ❑ Yes (exclude)

Section F

This paper reports on a…

Quantitative study ❑ Yes (continue screening)

Qualitative study ❑ Yes (continue screening)

Mixed methods study ❑ Yes (continue screening)

Research/review protocol ❑ Yes (exclude)

Systematic review and/or meta-analysis ❑ Yes (exclude)

Conference poster or abstract ❑ Yes (exclude)

Commentary or editorial ❑ Yes (exclude)

Grey literature (i.e., dissertation/ thesis) ❑ Yes (exclude)

Section G

Tick one of the following:

Include ❑

Exclude ❑

Unsure ❑

Comments/reasons:

| Code book | |
| --- | --- |
| Name of node (code) | Description |
| **Outcomes and measures** |  |
| ***Primary outcome*** | Code assigned to papers which specified a primary outcome. |
| ***Behaviours*** |  |
| Alcohol consumption | Relates to the frequency or duration of alcohol consumption, or the type of alcohol consumed. |
| Daily activities | Activities that are performed regularly as part of everyday life (e.g., chores or shopping). |
| Role limitations | The inability of an individual to perform their actual or perceived role in society due to physical or emotional problems. |
| Hair pulling |  |
| Meditation | An activity whereby people attempt to increase their awareness of the present moment by focusing their attention, usually on the breath, resulting in a more relaxed physical and psychological state. |
| Mindfulness | A state in which an individual attempts to remain present in the moment, acknowledging thoughts and feelings in a non-judgemental manner, which usually results in a more relaxed physical and psychological state. |
| Protective behaviours | Behaviours which can help to prevent condition onset or flare ups (e.g., hand washing, moisturiser use, wearing a hat). |
| Scratching | The behavioural response to itchy. |
| Sleep | Factors related to sleep hygiene (e.g., sleep quality, problems sleeping). |
| Smoking status |  |
| Treatment-related behaviours | Behaviours related to taking medication (i.e., treatment adherence or compliance). |
| Visit adherence | The rate of hospital visits. |
| ***Cognitions*** |  |
| Beliefs | Thoughts, views or opinions. |
| Cognitions | General cognitive processes that help people to make sense of their situation or surroundings. |
| Itch-related cognitions | Cognitive processes specifically related to the itch caused by a skin condition. |
| Concerns | A matter of interest or worry. |
| Knowledge | Improving knowledge. |
| Rumination | Repetitive and/or consistent thoughts about something. |
| Self-efficacy | The belief or confidence in one’s own ability to perform a behaviour or task. |
| ***Emotions*** |  |
| Affect | Feelings or emotions, which can be positive or negative. |
| Anxiety | Physical and/or psychological response to a stressor, which typically manifests as feelings of unease, worry or fear. |
| Depression | A state of low mood which lasts over time, which often results in a loss of pleasure in life or activities, feelings of hopelessness and in some more severe cases, suicidal ideation. |
| Distress | Typically, negative thoughts, feelings and behaviours that surmount to a person feeling overwhelmed and impact functioning. |
| Fear of cancer recurrence | Feeling afraid of cancer returning. |
| Shame | The feeling of humiliation or embarrassment in oneself. |
| Stress | A physical and psychological response to a perceived threat, which often occurs when an individual does not believe that they have the personal resources to overcome the perceived threat. |
| Worry | A state of feeling anxious or uneasy that results from thinking about a problem or challenge. |
| ***Other psychological factors*** |  |
| Psychological functioning | Composite measure of negative mood, depression and anxiety |
| Psychological inflexibility | One’s ability to adapt how they think and feel towards something, someone or a situation. |
| Psychological symptoms | A general term used to refer to symptoms relating to how we think and feel (e.g., feeling confused or sad). |
| Psychological well-being | A personal sense of satisfaction, contentment and potentially fulfilment with oneself or life. |
| Self-compassion | The ability to express compassion, kindness and understanding towards oneself. |
| Quality of life (QoL) | Personal perceptions of one’s position in life in relation to other factors such as social and cultural norms, personal goals, values and interests, health, economics, and the environment. |
| Improvement in QoL |  |
| Itch-related QoL |  |
| No improvement in QoL |  |
| ***Therapeutic relationship*** | Relationship between patients and practitioners |
| ***Social*** | This relates to societal issues, such as stigmatization, and social factors, including social support. |
| ***Physical*** | Physical aspects associated with having a skin condition (e.g., symptoms). |
| Body surface area |  |
| Disease severity |  |
| Fatigue |  |
| Itch |  |
| Physical functioning |  |
| Symptoms/severity worse |  |
| Symptoms/severity improved |  |
| No change in symptoms/severity |  |
| Change in symptoms/severity but not significant |  |
| **Study characteristics** |  |
| Conflict of Interest (COI) | A situation in which financial interests or other personal circumstances could influence a person’s professional judgement in conducting or reporting research. |
| No information provided |  |
| No COI declared |  |
| COI stated elsewhere |  |
| Eligibility criteria | Key requirements of participants for participation in a study. |
| Exclusion criteria |  |
| Inclusion criteria |  |
| Sampling approaches | The ways in which researchers select their study sample from the target population. |
| Convenience | Researchers approach individuals in a setting or location that is convenient and easy to reach the target population. |
| Multiple methods | Combination or two or more sampling approaches were used. |
| Purposive | A sample is selected based on the personal judgement of the researcher. |
| Unclear | Insufficient information to be able to decipher the sampling approach used. |
| Voluntary | Individuals from the target population volunteer to participate in research (e.g., an individual responds to a study advertisement they saw online). |
| Baseline measure |  |
| Follow up period | Points in time at which key outcomes were assessed. |
| Immediately after intervention |  |
| 1 month/4 weeks |  |
| 2 months/8 weeks |  |
| 3 months/12 weeks |  |
| 6 weeks |  |
| 6 months/24 weeks |  |
| 26 weeks |  |
| 11 months/48 weeks |  |
| 12 months/52 weeks |  |
| 60 weeks |  |
| **Intervention characteristics** |  |
| Assignments/homework | Tasks set for users to complete as part of the intervention. |
| Contact with patients | Users can communicate with other users via the intervention. |
| Contact with practitioner | Users can communicate with healthcare professionals via the intervention. |
| Provision of education/information | The intervention includes an educational component. |
| Reminders | Digital messages sent to prompt people to engage with the intervention. |
| Symptom tracking | The intervention allows users to monitor their symptoms (physical or psychological). |
| Treatment tracking | The intervention allows users to monitor treatment types and frequency/duration of use. |
| Tailored intervention | The intervention is fully or in part personalised to the individual user. |
| Medium of delivery | The type of digital technology used to deliver the intervention. |
| Combination of mediums | More than one type of digital technology used to deliver the intervention. |
| Mobile – text message | Text or SMS. |
| Mobile – application (app) |  |
| Online | Delivered via the internet. |
| *Pre-recorded video* |  |
| *Social media* |  |
| *Website* |  |
| Video conferencing platform/software | Synchronous (live) video technologies. |
| Nodes (Codes) for Qualitative Research | |
| **Acceptability** | How appropriate the intervention is perceived to be by the target audience. |
| Perceived effectiveness | Perceptions of how well the intervention achieves meets its purpose/achieves its goal. |
| Helpfulness | Perceptions relating to how helpful the intervention is. |
| Relevance | How suitable the intervention and its content is for the individual or group using it. |
| Design | How the intervention is presented and looks to the user. |
| **Satisfaction** | How satisfied people are with the intervention they receive. |
| **Feasibility** | How practical the intervention is perceived to be by the people receiving it. |
| **Facilitators** | Factors that are perceived to support intervention access or use. |
| Interactive activities | The intervention offers or requires users to complete tasks that require active engagement. |
| Reminders | Digital messages sent to prompt people to engage with the intervention. |
| **Barriers** | Factors that limit or prevent intervention use. |
| Individual factors | Factors that limit or prevent intervention use which are relevant to the individual user. |
| Daily routine/ activities | Other daily activities limit or prevent use of intervention. |
| Physical symptoms | Physical symptoms associated with a skin condition (e.g., fatigue, pain) |
| Not a personal priority | This relates to how important individuals perceive the intervention to be in comparison to other aspects of their life and what individuals are willing to make time for. |
| IT proficiency | Personal ability to use/navigate digital technology. |
| Lack of time | Individuals do not feel that they have time to use the intervention. |
| Personal preferences | Individual likes and dislikes relating to the design, content and function of the intervention. |
| Technical issues | Problems with the intervention technology (e.g., unable to log on or navigate the intervention, technology failures) |
| **Benefits/advantages** | Positives perceptions of the intervention and its use. |
| Facilitates acceptance | Individuals feel they are more accepting of themselves (e.g., body image) and/or their condition as a result of using the intervention. |
| Convenient | The intervention is easy to access, requires little effort and fits with the user’s personal needs or circumstances. |
| Facilitates emotional expression | Helps individuals to confront and convey their emotions. |
| Improves coping | Helps people to overcome challenges. |
| Improves well-being | Facilitates a sense of satisfaction, contentment and potentially fulfilment with one’s self or life. |
| Reduces isolation | Helps people to feel less isolated or alone. |
| Increases self-confidence | Trust in one’s own abilities or qualities. |
| Knowledge exchange | The intervention allows users to share knowledge of skin conditions and how to manage them. |
| Self-monitoring | Intervention users can track their own symptoms, feelings or treatment activities. |

# Sample sizes, attrition and participant demographics (gender and mean age)

| Study  (First author, year) | Number of participants | Number of dropouts | Females | Males | Mean age (SD) |
| --- | --- | --- | --- | --- | --- |
| Alinia 2017 (40) | 40 | 20 | 23 | 17 | 58.6 |
| Armstrong 2011 (41) | 80 | 3 | 36 | 44 | 48 (15) |
| Balato 2013 (42) | 40 | 0 | 18 | 22 | 38.8 (9.8) |
| Bundy 2013 (43) | 126 | 41 | 59 | 67 | 45.0 (12.6) |
| Domogalla 2021 (44) | 107 | 30 | 42 | 65 | 49.1 (12.1) |
| Erdil 2020 (45) | 81 | 41 | 37 | 44 | 37.1 (11.8) |
| Hawkins 2016 (46) | 50 | 28 | Not stated | Not stated | Not stated |
| Heckman 2021 (47) | 137 | 30 | 56 | 44 | 40.9 |
| Hedman-Lagerlöf 2021 (48) | 102 | 11 | 83 | 19 | 37 (11) |
| Iliffe 2019 (49) | 12 | N/A | 12 | 0 | Not stated |
| Joergensen 2020 (50) | 83 | Unclear | 59 | 24 | Not stated |
| Koulil 2018 (51) | 2 | N/A | 1 | 1 | 45 |
| Lee 2018 (52) | 22 | 10 | 19 | 3 | Group 1 (n = 12) 34.2 (9.4). Group 2 (n = 10) 30.4 (6.8) |
| Manne 2021 (53) | 441 | 59 | 216 | 225 | 61.4 (13.3) |
| Marasca 2020 (54) | 23 | N/A | 13 | 10 | 35.4 |
| Mollerup 2016 (55) | 140 | N/A | 90 | 50 | 38.1 (13.7) |
| Russell 2019 (56) | 69 | 37 | 37 | 32 | IG (n = 46) 53.5 (12.1)  CG (n = 23) 53.1 (15.2) |
| Schuster 2020 (57) | 101 | N/A | 73 | 28 | 47.4 (15.4) |
| Sherman 2019 (58) | 50 | 0 | 35 | 15 | 27.1 (10.8) |
| Svendsen 2018 (59) | 134 | 12 | 82 | 52 | 48 |
| Van Beugen 2016 (60) | 131 | 49 | 64 | 67 | 53.1 (12.3) |
| Van Cranenburgh 2015 (61) | 11 dermatologists, 105 patients | 35 patients | UC |  | Not provided |
| Zhao 2020 (62) | 221 | 180 | 100 | 121 | 34.1 (10.6) |
| TOTAL | 2,268 | 556 | 1,132 | 933 | - |
| N/A, not applicable. | | | | | |

# Outcome variables and measurement tools

| Study | Category | Outcome | Measure | Additional Information |
| --- | --- | --- | --- | --- |
| Alinia 2017 (40) |  |  |  |  |
|  | Behavioural | Alcohol consumption | Unclear |  |
|  | Behavioural | Smoking status | Unclear | Included in analysis as a covariate |
|  | Behavioural | Treatment adherence | MEMS® caps | Electronic Monitoring System |
|  | Physical | Disease severity | PASI |  |
|  | Physical | Disease severity | IGA |  |
| Armstrong 2011 (41) |  |  |  |  |
|  | Cognitive | Knowledge of condition | 14-item questionnaire | Questions based on intervention content |
|  | Physical | Disease severity | POEM |  |
|  | Satisfaction | Satisfaction with intervention (appeal and usefulness) | 10-point rating scale |  |
| Balato 2013 (42) |  |  |  |  |
|  | Other psychological | Quality of life | DLQI |  |
|  | Behavioural | Treatment adherence  (number of days per week medication forgotten) | One item | MCQ |
|  | Behavioural | Treatment adherence  (number of days medication taken) | Seven-day calendar |  |
|  | Physical | Disease severity | PASI |  |
|  | Physical | Disease severity | SAPASI |  |
|  | Physical | Disease severity | PGA |  |
|  | Physical | Disease severity | BSA |  |
|  | Therapeutic relationship | Patient-physician relationship | Questionnaire including a 10-point rating scale |  |
| Bundy 2013 (43) |  |  |  |  |
|  | Cognitive | Beliefs about condition | IPQ-R |  |
|  | Other psychological | Anxiety | HADS |  |
|  | Other psychological | Depression | HADS |  |
|  | Other psychological | Quality of life | DLQI |  |
|  | Behavioural | Alcohol consumption | Unclear |  |
|  | Behavioural | Smoking status | Unclear | Included in analysis as a predictor variable |
|  | Physical | Disease severity | SAPASI |  |
| Domogalla 2021 (44) |  |  |  |  |
|  | Emotional | Mood | Likert scale |  |
|  | Other psychological | Quality of life | DLQI |  |
|  | Other psychological | Anxiety | HADS |  |
|  | Other psychological | Depression | HADS |  |
|  | Behavioural | Alcohol consumption  (number of days alcohol consumed per week) | One item |  |
|  | Behavioural | Smoking status | Unclear | Measured for demographic purposes only |
|  | Behavioural | Daily activities | Likert scale |  |
|  | Physical | Itch | Numeric rating scale |  |
|  | Physical | Disease severity | PASI |  |
| Erdil 2020 (45) |  |  |  |  |
|  | Cognitive | Knowledge of condition | Eczema assessment questionnaire | 20 true/false items on preventative measures |
|  | Behavioural | Protective behaviours  (Hand washing and use of moisturizer, gloves, liquid soap, cologne, wet wipes) | Scores were based on the irritation exposure score developed by Jungbauer. | Use of cologne, wet wipes, moisturizers, and gloves, were added to the scoring system. |
|  | Behavioural | Treatment compliance  (frequency of medication use) | Unclear |  |
|  | Physical | Disease severity | HECSI |  |
|  | Physical | Disease severity | 5-point scale VAS |  |
| Hawkins 2017 (46) |  |  |  |  |
|  | Cognitive | Knowledge of condition | Unclear |  |
|  | Cognitive | Treatment concerns  (extent to which the clinician addressed patient concerns) | VAS |  |
|  | Behavioural | Treatment adherence | One item | MCQ |
|  | Satisfaction | Satisfaction with intervention | Unclear |  |
| Heckman 2021 (47) |  |  |  |  |
|  | Cognitive | Itch cognitions | Itch cognitions questionnaire |  |
|  |  | Perceived Efﬁcacy in Patient-Physician Interactions | PEPPI-5 |  |
|  | Emotional | Stress | PSS |  |
|  | Other psychological | Itch-related quality of life | ItchyQoL questionnaire |  |
|  | Behavioural | Scratch | Sleep-Related Itch and Scratch |  |
|  | Behavioural | Scratch | Scratch Intensity and Impact Scale |  |
|  | Behavioural | Risk of treatment non-adherence | MMAS-8-IS | 8-item scale |
|  | Physical | Itch | 5-D Pruritus Scale |  |
|  | Physical | Itch severity and scratching pleasure | 10-point numeric rating scale |  |
|  | Intervention usage | Frequency and duration of intervention use |  |  |
|  | Intervention usage | Completion rate |  |  |
| Hedman-Lagerlöf  2021 (48) |  |  |  |  |
|  | Emotional | Stress | PSS |  |
|  | Other psychological | Anxiety | Beck Anxiety Inventory |  |
|  | Other psychological | Depression | PHQ-9 |  |
|  | Other psychological | Quality of life | DLQI |  |
|  | Other psychological | Quality of life | BBQ |  |
|  | Behavioural | Sleep problems | ISI |  |
|  | Physical | Itch | 5-D Pruritus Scale |  |
|  | Physical | itch intensity (past 48 hours) | VAS |  |
|  | Physical | Disease severity | POEM |  |
|  | General health | Health status | One item |  |
|  | Satisfaction | Satisfaction with intervention | CSQ |  |
| Iliffe 2019 (49) |  |  |  |  |
|  | Qualitative | Personal experiences of an online support group for people living with alopecia | Semi-structured interviews |  |
| Joergensen 2020 (50) |  |  |  |  |
|  | Other psychological | Quality of life | DLQI |  |
|  | Behavioural | Treatment adherence | Clicks of memory button |  |
|  | Behavioural | Treatment adherence | Manual tracking via a mobile app |  |
|  | Physical | Disease severity | POEM |  |
|  | Physical | Disease severity | EASI |  |
|  | Physical | Disease severity | SCORAD |  |
| Koulil 2018 (51) |  |  |  |  |
|  | Cognitive | Illness cognitions | Illness cognitions questionnaire | Helplessness and acceptance subscales only |
|  | Emotional | Worry | PSWQ |  |
|  | Other psychological | Anxiety | ISDL |  |
|  | Other psychological | Depression | Beck Depression Inventory |  |
|  | Behavioural | Sleep hygiene | A sleep schedule |  |
|  | Behavioural | Scratch | ISDL | Conscious and automatic scratching sub-scales |
|  | Behavioural | Treatment compliance  (frequency of medication use) | Questionnaire | 5-point Likert scale |
|  | Physical | Disease severity | PASI |  |
|  | Physical | Disease severity | SAPASI |  |
|  | Therapeutic relationship | Patient-physician relationship | WAI-S |  |
|  | Therapeutic relationship | Patient-physician relationship | ITRQ |  |
|  | Therapeutic relationship | How useful/motivating patient found contact with the therapist | Numeric rating scale | 6-point |
|  | Satisfaction | Satisfaction with intervention | Unclear |  |
|  | Impact on daily life | Role limitations due to physical and emotional problems | RAND-36 Health Status Inventory |  |
|  | Social | Perceived social support | ISDL |  |
|  | Social | Stigmatization | ISDL |  |
| Lee 2018 (52) |  |  |  |  |
|  | Emotional | Shame | ESS |  |
|  | Other psychological | Quality of life | QOLS |  |
|  | Other psychological | Psychological inflexibility | AAQ-TTM |  |
|  | Other psychological | Psychological symptoms | MINI |  |
|  | Behavioural | Hair pulling | MGH-HPS |  |
|  | Therapeutic relationship | Patient-physician relationship | WAI-SR |  |
|  | Satisfaction | Satisfaction with intervention | CSQ-8 |  |
| Manne 2021 (53) |  |  |  |  |
|  | Cognitive | Knowledge of condition | 13-item questionnaire | True/false questions on melanoma |
|  | Cognitive | Knowledge of condition | 6-item questionnaire | MCQ on characteristics of abnormal lesions |
|  | Cognitive | Self-efficacy for performing a skin self-examination | 12-item questionnaire |  |
|  | Cognitive | Self-efficacy for performing sun protection behaviours | Four Likert scale items |  |
|  | Behavioural | Self-management issues (use of gloves, topical steroids, consulting General Practitioner) | 10-item questionnaire | Questionnaire developed for the study. |
|  | Behavioural | Frequency of sun protection behaviours (use of sunscreen, hats, long-sleeves, and staying in the shade). | Four items comprised of 5-point Likert scales |  |
|  | Behavioural | Performance of a skin self-examination | One item | Yes/no response |
|  | Intervention usage | Number of page visits |  |  |
|  | Intervention usage | Completion rate |  |  |
|  | Acceptability | Barriers (technological, personal and general barriers to use, and intervention-specific barriers) | 22-item questionnaire |  |
|  | Acceptability | Perceived effectiveness of intervention | 20-item Impact and Effectiveness measure |  |
|  | Acceptability | Programme characteristics (usefulness, convenience, ease of use and navigation, worry about privacy, and satisfaction) | 15-item Evaluation and Utility survey based on 5-point Likert scales |  |
| Marasca 2020 (54) |  |  |  |  |
|  | Other psychological | Quality of life | DLQI |  |
|  | Other psychological | Psychological well-being | PGWB |  |
| Mollerup 2016 (55) |  |  |  |  |
|  | Cognitive | Self-efficacy | 10-point numeric rating scale |  |
|  | Other psychological | Quality of life | DLQI |  |
|  | Behavioural | Treatment adherence | DMARS-4 |  |
|  | Physical | Itch (in the past 4 weeks) | VAS |  |
|  | Physical | Disease severity | HECSI |  |
|  | Physical | Disease severity | 10-point VAS |  |
|  | Intervention usage | Number of page visits |  |  |
| Schuster 2020 (57) |  |  |  |  |
|  | Emotional | Affect | Scale of Positive and Negative Experience |  |
|  | Emotional | Happiness | One item | Taken from European Social Survey |
|  | Emotional | Facebook envy | 6-item scale |  |
|  | Other psychological | Quality of life | DLQI |  |
|  | Satisfaction | Life satisfaction | Satisfaction with Life Scale |  |
|  | Intervention usage | Frequency and duration of intervention use |  |  |
| Sherman 2019 (58) |  |  |  |  |
|  | Emotional | Affect | PANAS |  |
|  | Emotional | Body image related distress | Body Image Disturbance Questionnaire |  |
|  | Other psychological | Self-compassion | SCS-SF |  |
|  | Physical | Disease severity | One item (5-point Likert scale) |  |
| Svendsen 2018 (59) |  |  |  |  |
|  | Other psychological | Quality of life | DLQI |  |
|  | Behavioural | Smoking status | Unclear | Included as a covariate |
|  | Behavioural | Treatment adherence (PO) | Electronic medication dispensers |  |
|  | Behavioural | Treatment adherence (PO) | Weight of medication canisters | Weighed before/after use |
|  | Physical | Disease severity | PASI |  |
|  | Physical | Disease severity | LS-PGA |  |
| Russell 2019 (56) |  |  |  |  |
|  | Cognitive | Rumination | Rumination and Reflection questionnaire | Rumination subscale |
|  | Emotional | Stress | PSS |  |
|  | Emotional | Worry | PSQW-A |  |
|  | Emotional | Fear of cancer recurrence | FCRI |  |
|  | Other psychological | Experience of mindfulness | CAMS-R |  |
|  | Behavioural | Frequency of informal meditation practice | 3-item questionnaire |  |
|  | Acceptability | Relevance of intervention content (benefits and participant preferences) | Three open-ended items | Completed each week |
|  | Acceptability | Perceived helpfulness of intervention | Three items | Completed post-intervention. |
| van Beugen 2016 (60) |  |  |  |  |
|  | Behavioural | Treatment compliance | Unclear |  |
|  | Other psychological | Psychological functioning (depression) | Beck Depression Inventory | Composite measure of psychological functioning including measures of negative mood, depression, and anxiety |
|  | Other psychological | Psychological functioning (negative mood and anxiety) | ISDL |  |
|  | Physical | Disease severity | PASI |  |
|  | Physical | Disease severity | SAPASI |  |
|  | Physical | Physical functioning (itch) | ISDL | Composite measure of physical functioning including itch and fatigue. 4-item subscale for itch |
|  | Impact on daily life | Role limitations | RAND-36 Health Status Inventory |  |
| van Cranenburgh 2015 (61) |  |  |  |  |
|  | Intervention usage | Frequency and duration of use |  |  |
|  | Intervention usage | Number of page visits |  |  |
|  | Feasibility | Factors preventing intervention use | 2 Likert scale items |  |
|  | Acceptability | Relevance of intervention | 10 items based on 5-point Likert scale |  |
|  | Acceptability | Convenience of intervention | 4 items based on 5-point Likert scale |  |
|  | Acceptability | Intervention design  (layout and attractiveness) | 5 items based on 5-point Likert scale |  |
|  | Acceptability | Intervention design  (font size and text length) | 2 items based on 5-point Likert scale |  |
| Zhao 2020 (62) |  |  |  |  |
|  | Behavioural | Treatment adherence | Questionnaire |  |
|  | Behavioural | Visit adherence | Rate of patient hospital visits |  |
| MEMS, Medication Event Monitoring System; PASI, Psoriasis Area and Severity Index; IGA, Investigator’s Global Assessment; POEM, Patient Oriented Eczema Measure; DLQI, Dermatology Life Quality Index; SAPASI, Self-Administered Psoriasis Area and Severity Index; PGA, Physician’s Global Assessment; LS-PGA, Lattice System Physician’s Global Assessment; BSA, Body Surface Area; IPQ-R, Illness Perception Questionnaire Revised; HADS, Hospital Anxiety Depression Scale; HECSI, Hand Eczema Clinical Severity Index; PEPPI-5, Perceived Efﬁcacy in Patient-Physician Interactions; PSS, Perceived Stress Scale; MMAS-8-IS, Morisky Medication Adherence Scale Itch Speciﬁc; PHQ-9, Patient Health Questionnaire 9; BBQ, Brunnsviken Brief Quality of Life Scale; ISI, Insomnia Severity Index; CSQ, Client Satisfaction Questionnaire; CSQ-8, Client Satisfaction Questionnaire–8; EASI, Eczema Area and Severity Index; SCORAD, SCORing Atopic Dermatitis; PSWQ, Penn State Worry Questionnaire; PSQW-A, Penn State Worry Questionnaire-Abbreviated; ISDL, Impact of Chronic Skin Disease on Daily Life; ITRQ, Internet-Specific Therapeutic Relationship Questionnaire; ESS, Experience of Shame Scale; QOLS, Quality of Life Scale; AAQ-TTM, Acceptance and Action Questionnaire for Trichotillomania; MINI, The Miniature International Neuropsychiatric Interview; MGH-HPS, Massachusetts General Hospital Hair Pulling Scale; WAI-SR, Working Alliance Inventory – Short Revised; PGWB, Psychological-General-Well-Being-Index; DMARS-4, Danish Medication Adherence Report Scale; PANAS, Positive and Negative Aﬀect Schedule; SCS-SF, 12-item Self-Compassion Scale Short Form; FCRI, Fear of cancer recurrence Inventory; CAMS-R, Cognitive and Affective Mindfulness Scale-Revised; | | | | |

# Table 3. Critical appraisal of methodological quality

| Study  (first author, year) | Q1 | Q2 | Q3 | Q4 | Q5 | Q6 | Q7 | Q8 | Q9 | Q10 | Q11 | Q12 | Q13 | Total Score |
| --- | --- | --- | --- | --- | --- | --- | --- | --- | --- | --- | --- | --- | --- | --- |
| RCTs^1^ | | | | | | | | | | | | | | |
| Alinia 2017 (40) | UC | UC | Y | UC | UC | UC | Y | Y | Y | Y | Y | Y | Y | 8/13 |
| Armstrong 2011 (41) | Y | Y | Y | Y | UC | N | N | N | Y | Y | UC | Y | Y | 8/13 |
| Balato 2013 (42) | Y | Y | Y | N | Y | Y | Y | UC | Y | N/A | Y | Y | Y | 10/12 |
| Bundy 2013 (43) | Y | UC | Y | N | N/A | N/A | Y | Y | Y | Y | Y | Y | Y | 9/11 |
| Domogalla 2021 (44) | UC | Y | Y | N | N | UC | Y | Y | UC | Y | N | UC | Y | 6/13 |
| Erdil 2020 (45) | Y | N | Y | N | N | N | Y | Y | Y | Y | UC | Y | Y | 8/13 |
| Hawkins 2016 (46) | Y | UC | UC | UC | N | UC | UC | N | Y | UC | UC | Y | Y | 4/13 |
| Hedman-Lagerlöf 2021 (48) | Y | Y | Y | Y | UC | UC | Y | N | Y | Y | Y | Y | Y | 10/13 |
| Joergensen 2020 (50) | Y | UC | Y | N | Y | UC | UC | UC | Y | Y | Y | Y | Y | 8/13 |
| Lee 2018 (52) | Y | UC | Y | N | N | N | Y | N | Y | Y | Y | Y | Y | 8/13 |
| Manne 2021 (53) | Y | UC | Y | N | N | N | Y | Y | Y | Y | Y | UC | Y | 8/13 |
| Mollerup 2016 (55) | UC | UC | Y | UC | UC | UC | N | N | Y | Y | Y | UC | UC | 4/13 |
| Russell 2019 (56) | Y | Y | Y | N | N/A | UC | Y | Y | Y | Y | Y | Y | Y | 10/12 |
| Sherman 2019 (58) | Y | UC | N | Y | N/A | UC | Y | Y | N | Y | UC | UC | Y | 6/12 |
| Svendsen 2018 (59) | Y | Y | Y | N | N | N | Y | N | Y | Y | Y | Y | Y | 9/13 |
| van Beugen 2016 (60) | Y | Y | UC | N | N | N | Y | Y | Y | Y | UC | Y | Y | 8/13 |
| Zhao 2020 (62) | Y | UC | N | UC | N | UC | Y | N | UC | Y | UC | Y | Y | 5/13 |
| Quasi-experimental studies^2^ | | | | | | | | | | | | | | |
| Marasca 2020 (54) | Y | N/A | N/A | N | Y | N/A | N/A | N/A | N | - | - | - | - | 2/4 |
| Analytical cross-sectional studies^3^ | | | | | | | | | | | | | | |
| Schuster 2020 (57) | N | Y | Y | UC | UC | N | Y | UC | - | - | - | - | - | 3/8 |
| Van Cranenburgh 2015 (61) | Y | Y | UC | N/A | UC | N | Y | Y | - | - | - | - | - | 4/7 |
| Case reports^4^ | | | | | | | | | | | | | | |
| Koulil 2018 (51) | N | N | N | Y | Y | Y | N | Y | - | - | - | - | - | 4/8 |
| Cohort studies^5^ | | | | | | | | | | | | | | |
| Heckman 2021 (47) | N/A | N/A | Y | Y | Y | Y | Y | Y | N | N | Y | - | - | 8/9 |
| Qualitative research^6^ | | | | | | | | | | | | | | |
| Iliffe 2019 (49) | Y | Y | Y | Y | Y | N | N | Y | Y | Y | - | - | - | 8/10 |
| Mollerup 2016 (55) | UC | Y | Y | Y | Y | N | N | UC | Y | Y | - | - | - | 6/10 |
| RCTs, Randomised Controlled Trials; N, no; Y, yes; UC, unclear; N/A, not applicable | | | | | | | | | | | | | | |
| ^1^JBI checklist questions for RCTs (33): 1) Was true randomization used for assignment of participants to treatment groups?; 2) Was allocation to treatment groups concealed?; 3) Were treatment groups similar at the baseline?; 4) Were participants blind to treatment assignment?; 5) Were those delivering treatment blind to treatment assignment?; 6) Were outcomes assessors blind to treatment assignment?; 7) Were treatment groups treated identically other than the intervention of interest?; 8) Was follow up complete and if not, were differences between groups in terms of their follow up adequately described and analyzed?; 9) Were participants analyzed in the groups to which they were randomized?; 10) Were outcomes measured in the same way for treatment groups?; 11) Were outcomes measured in a reliable way?; 12) Was appropriate statistical analysis used?; 13) Was the trial design appropriate, and any deviations from the standard RCT design (individual randomization, parallel groups) accounted for in the conduct and analysis of the trial? | | | | | | | | | | | | | | |
| ^2^JBI checklist questions for quasi-experimental studies (33): 1) Is it clear in the study what is the ‘cause’ and what is the ‘effect’ (i.e. there is no confusion about which variable comes first)?; 2) Were the participants included in any comparisons similar?; 3) Were the participants included in any comparisons receiving similar treatment/care, other than the exposure or intervention of interest?; 4) Was there a control group?; 5) Were there multiple measurements of the outcome both pre and post the intervention/exposure?; 6) Was follow up complete and if not, were differences between groups in terms of their follow up adequately described and analyzed?; 7) Were the outcomes of participants included in any comparisons measured in the same way?; 8) Were outcomes measured in a reliable way?; 9) Was appropriate statistical analysis used? | | | | | | | | | | | | | | |
| ^3^JBI checklist questions for analytical cross-sectional studies (34): 1) Were the criteria for inclusion in the sample clearly defined?; 2) Were the study subjects and the setting described in detail?; 3) Was the exposure measured in a valid and reliable way?; 4) Were objective, standard criteria used for measurement of the condition?; 5) Were confounding factors identified?; 6) Were strategies to deal with confounding factors stated?; 7) Were the outcomes measured in a valid and reliable way?; 8) Was appropriate statistical analysis used? | | | | | | | | | | | | | | |
| ^4^JBI checklist questions for case reports (34): 1) Were patient’s demographic characteristics clearly described?; 2) Was the patient’s history clearly described and presented as a timeline?; 3) Was the current clinical condition of the patient on presentation clearly described?; 4) Were diagnostic tests or assessment methods and the results clearly described?; 5) Was the intervention(s) or treatment procedure(s) clearly described?; 6) Was the post-intervention clinical condition clearly described?; 7) Were adverse events (harms) or unanticipated events identified and described?; 8) Does the case report provide takeaway lessons? | | | | | | | | | | | | | | |
| ^5^JBI checklist questions for cohort studies (34): 1) Were the two groups similar and recruited from the same population?; 2) Were the exposures measured similarly to assign people to both exposed and unexposed groups?; 3) Was the exposure measured in a valid and reliable way?; 4) Were confounding factors identified?; 5) Were strategies to deal with confounding factors stated?; 6) Were the groups/participants free of the outcome at the start of the study (or at the moment of exposure)?; 7) Were the outcomes measured in a valid and reliable way?; 8) Was the follow up time reported and sufficient to be long enough for outcomes to occur?; 9) Was follow up complete, and if not, were the reasons to loss to follow up described and explored?; 10) Were strategies to address incomplete follow up utilized?; 11) Was appropriate statistical analysis used? | | | | | | | | | | | | | | |
| ^6^JBI checklist questions for qualitative research (35): 1) Is there congruity between the stated philosophical perspective and the research methodology?; 2) Is there congruity between the research methodology and the research question or objectives?; 3) Is there congruity between the research methodology and the methods used to collect data?; 4) Is there congruity between the research methodology and the representation and analysis of data?; 5) Is there congruity between the research methodology and the interpretation of results?; 6) Is there a statement locating the researcher culturally or theoretically?; 7) Is the influence of the researcher on the research, and vice- versa, addressed? 8) Are participants, and their voices, adequately represented?; 9) Is the research ethical according to current criteria or, for recent studies, and is there evidence of ethical approval by an appropriate body?; 10) Do the conclusions drawn in the research report flow from the analysis, or interpretation, of the data? | | | | | | | | | | | | | | |

# Table 4. JBI levels of evidence for effectiveness and meaningfulness (37)

| Level of evidence | Study design | | *n* | Study (first author, year) |
| --- | --- | --- | --- | --- |
|  | Effectiveness | |  |  |
| 1: Experimental designs | 1a | Systematic review of RCTs | N/A | - |
|  | 1b | Systematic review of RCTs and other study designs | N/A | - |
|  | 1c | RCT | 12 | Armstrong 2011 (41) |
|  |  |  |  | Balato 2013 (42) |
|  |  |  |  | Bundy 2013 (43) |
|  |  |  |  | Domogalla 2021 (44) |
|  |  |  |  | Erdil 2020 (45) |
|  |  |  |  | Hedman-Lagerlöf 2021 (48) |
|  |  |  |  | Manne 2021 (53) |
|  |  |  |  | Russell 2019 (56) |
|  |  |  |  | Sherman 2019 (58) |
|  |  |  |  | Svendsen 2018 (59) |
|  |  |  |  | van Beugen 2016 (60) |
|  |  |  |  | Zhao 2021 (62) |
|  | 1d | Pseudo-RCTs | 3 | Lee 2018 (52) |
|  |  |  |  | Hawkins 2017 (46) |
|  |  |  |  | Joergensen 2020 (50) |
|  | 1 unclear* | Unable to assign to a specific level due to limited information |  | Alinia 2017 (40)  Mollerup 2016 (55) |
| 2: Quasi-experimental designs | 2a | Systematic review of quasi-experimental studies | N/A | - |
|  | 2b | Systematic review of quasi-experimental studies and other lower study designs | N/A | - |
|  | 2c | Quasi-experimental studies prospectively controlled study | 0 | - |
|  | 2d | Pre-test – post-test or historic/retrospective control group study | 2 | Heckman 2021 (47)  Marasca 2020 (54) |
| 3: Observational – analytical designs | 3a | Systematic review of comparable cohort studies | N/A | - |
|  | 3b | Systematic review of comparable cohort studies and other lower study designs | N/A | - |
|  | 3c | Cohort study with control group | 0 | - |
|  | 3d | Case-controlled study | 0 | - |
|  | 3e | Observational study without a control group. | 1 | Van Cranenburgh 2015 (61) |
| 4: Observational – descriptive studies | 4a | Systematic review of descriptive studies | N/A | - |
|  | 4b | Cross-sectional study | 1 | Schuster 2020 (57) |
|  | 4c | Case series | 0 | - |
|  | 4d | Case study | 1 | Koulil 2018 (51) |
| 5: Expert opinion and bench research | 5a | Systematic review of expert opinion | N/A | - |
|  | 5b | Expert consensus | N/A | - |
|  | 5c | Bench research, single expert opinion | N/A | - |
|  |  | Meaningfulness |  |  |
| 1 |  | Qualitative or mixed-methods systematic review | N/A | - |
| 2 |  | Qualitative or mixed-methods synthesis | N/A | - |
|  |  |  |  |  |
| 3 |  | Single qualitative study | 2 | Iliffe 2019 (49)  Mollerup 2016 (55) |
| 4 |  | Systematic review of expert opinion | N/A | - |
| 5 |  | Expert opinion | N/A | - |
| RCTs, Randomised Controlled Trial; N/A, not applicable (to this systematic review) *We added this category for papers with limited or contradictory information, which prevented assignment to a specific level. | | | |  |

# Intervention characteristics according to the Template for Intervention Description and Replication (TIDieR) checklist and guide

| TIDieR item | Relevant information |
| --- | --- |
| Alinia 2017 (40) |  |
| 1 | Online psoriasis symptom questionnaire. |
| 2 | Aimed to investigate the effectiveness of weekly online symptom reporting for maintaining treatment adherence. |
| 3 & 4 | Participants were prescribed Fluocinonide (0.05%) (topical medication) with an electronic cap that tracked medication use. They were given a treatment protocol at the initial study visit and were instructed to refill their medication canister when empty. Clinic visits were scheduled for 1, 3, 6 and 12 months after initial appointments. Participants reported their symptoms online on a weekly basis, but no detail on the questionnaire provided. |
| 5 | The intervention was for people living with psoriasis and was provided by study staff. No mention of staff training. |
| 6 | The intervention was designed for individual use. No information provided on the online questionnaire for symptom tracking. Medication Event Monitoring System (MEMS) recorded the number of times participants opened their medication. |
| 7 | No information provided on location of visits after recruitment from Wake Forrest Medical Centre, (Winston-Salem, USA). |
| 8 | Participants were prescribed Fluocinonide twice daily and recorded psoriasis symptoms weekly online. One initial visit plus four follow up visits scheduled over 12-month study period. |
| 9 | Participants had the ability to track their own symptoms. |
| 10 | None described. |
| 11 & 12 | 11) 20/40 participants completed the study. Half withdrew early due to worsening psoriasis (need for change of treatment) (n = 3), starting a concomitant medication (n = 3), not liking the medication (n = 4), burning side-effects (n = 1), loss to follow-up (n = 8) and fungal infection (n = 1). One cap did not record any data. 12) No data on questionnaire completion rates. |
| Armstrong 2011 (41) |  |
| 1 | Online educational video. |
| 2 | Aimed to improve knowledge of atopic dermatitis and skin care for atopic dermatitis, plus disease severity. |
| 3 & 4 | The online video contained information on the clinical manifestations of atopic dermatitis, contributing environmental factors, bathing and hand washing techniques, moisturizer vehicles, and common treatment modalities. Participants received instructions for video access and use and were asked to demonstrate how to locate and view the video during the initial clinic visit. |
| 5 | The intervention was for people with atopic dermatitis. It was not clear who created the video content. Intervention provider training was not applicable. |
| 6 | The intervention was designed for individual use. Participants accessed the video online using the instructions provided. |
| 7 | Participants attended an initial visit to a dermatology clinic but the intervention was delivered online. |
| 8 | Participants were instructed to view the educational material at least once during the 12-week study period. Patients could review the material as often as they desired after the initial viewing. |
| 9 | This intervention was not tailored to individual users. The video included generic information on atopic dermatitis and care. |
| 10 | None described. |
| 11 & 12 | 11) The Flesch-Kincaid readability score of the pamphlet was assessed to be 46.06 with a reading level closest to 13- to 15-year-olds, but participants health literacy level was not assessed. 12) The number of participants in both arms of the trial that were lost to follow up were reported, but reasons for drop out were not. |
| Balato 2013 (42) |  |
| 1 | The following names were used to refer to the intervention: educational and motivational support service; mobile-phone-based intervention; and text message intervention. |
| 2 | Aimed to evaluate the use of text messaging in improving treatment adherence and several patient outcomes such as quality of life, disease severity, patient-perceived disease severity and the patient–physician relationship. |
| 3 & 4 | Treatment reminders and educational messages were sent to participants via text message. Text messages were written using simple language, considering frequently asked questions about psoriatic drugs (e.g., adverse effects, administration) and general recommendations for overall health. Patients also received a 7-day calendar to track adherence. Intervention provider training was not applicable. |
| 5 | The study investigators were physicians and were responsible for sending the text messages to people with psoriasis. |
| 6 | Treatment reminders and educational messages were sent to participants s’ mobile phones via text message. |
| 7 | Patients were required to have access to a cellular phone that was capable of receiving text messages. |
| 8 | Participants received seven text messages per week (1 per day) for a period of 12 weeks in a randomly selected order (reminders three times weekly and educational tools four times weekly). |
| 9 | Text messages were not tailored to individual users. |
| 10 | None described. |
| 11 & 12 | Not described or reported on. |
| Bundy 2013 (43) |  |
| 1 | Electronic Targeted Intervention for Psoriasis (eTIPs) treatment programme. |
| 2 | Aimed to determine if eTIPs would reduce distress, improve quality of life and clinical severity in patients with psoriasis. |
| 3 & 4 | A programme comprised of six modules of Cognitive Behavioural Therapy plus information on: management of self-esteem; thinking styles; low mood and depression; stress and tension; enhancing coping; and information about psoriasis its general management. After login, patients read module material, listened to simulated patients (actors) talk about common experiences and completed brief assignments designed to test and reinforce understanding of core concepts. Intervention provider training was not applicable. |
| 5 | The intervention was intended for people with psoriasis. It was developed by the research team, but no information on the expertise or background of the individual team members was provided. |
| 6 | The intervention was designed for individual use. |
| 7 | Participants accessed the intervention online. |
| 8 | Participants could choose when to complete the modules but were encouraged to complete at least 1 module per week. |
| 9 | The educational content was specific to psoriasis but was not tailored for individual users. |
| 10 | None described. |
| 11 & 12 | The CBT modules that formed part of the eTIPs programme followed gold-standard CBT protocols for face-to-face delivery in other health conditions. The rate of attrition rate was recorded. All patients completed at least one course module. In total, 76–85% (depending on the outcome) provided usable post-treatment data. At follow-up, five people from the withdrew from the treatment group. Attrition rate: 41 participants (32%) did not complete the study, including 26 people (43%) from the intervention group and 15 people (23%) from the control group. |
| Domogalla 2021 (44) |  |
| 1 | Educational programme plus a disease management eHealth smartphone app/ psoriasis monitoring app called DermaScope Mobile. |
| 2 | Rationale: eHealth devices have shown positive effects on common chronic diseases, including diabetes, hypertension, chronic heart failure, and asthma, yet data are limited in psoriasis. An educational programme developed by Bubak et al. (2019) improved knowledge in psoriasis and general health, but not mental health. This study aimed to improve the mental health of patients with psoriasis. |
| 3 & 4 | A 2-hour educational programme on psoriasis pathogenesis, therapy options, and comorbidities. Participants attended baseline visits. The intervention group attended the educational programme and after received a personal anonymized access code and an introduction to the psoriasis monitoring app, which allowed for regular photodocumentation of the skin. Participants tracked their quality of life, mood, activity, pain, and pruritus by completing health questionnaires within the app, and could freely contact specialized dermatologists unrestrictedly via a chat feature in the app. |
| 5 | The intervention is intended for people with psoriasis. Dermatology specialists delivered the educational program. |
| 6 | The intervention was designed for individual use. A combination of in-person and remote delivery methods were used; the educational program was held in person in a group setting before individuals could access the smartphone app. |
| 7 | The group education session was delivered at an outpatient dermatology clinic within the Department of Dermatology, Venereology, and Allergology at the University Medical Center Mannheim, Germany. Participants accessed the app via their personal smartphone using their own anonymized access code. |
| 8 | Participants were asked to photo-document their skin condition for 60 weeks and input other health data to the app once per week. |
| 9 | Participants monitored their condition, quality of life, mood, activity, pain, and pruritus via the app. They were able to contact a dermatologist via the app, suggesting participants could receive tailored advice. |
| 10 | None described. |
| 11 & 12 | The authors assessed and reported the frequency of app use, as well as the rate of and reasons for dropouts. 20% (10/49) of participants in the control group and 19% (9/47) of participants in the intervention group discontinued use by week 60. Reported reasons for withdraw included: a lack of time (12/107, 11.2%); unavailability or nonappearance (10/107, 9.3%); distance to the outpatient clinic or relocating (4/107, 3.7%); poor health status (2/107, 1.9%); and other reasons (3/107, 2.8%). |
| Erdil 2020 (45) |  |
| 1 | The following names were used to refer to the intervention: short message service (SMS) and text messaging (TM). |
| 2 | Aimed to determine whether regular SMS reminder and information messages improve the medication adherence and knowledge of patients with hand eczema. |
| 3 & 4 | A new module was created on the dermatology patient education website, DermPatientEd.com, to display an educational video about psoriasis, text-based information, and graphics about side effects. A research fellow recruited clinic patients to the study. Participants received a paper card with a link to the intervention (module and survey) or control (survey only) conditions. Patients viewed the educational content and completed the survey online. |
| 5 | Research fellow was responsible for recruiting people with psoriasis to join the study. Participants were examined by doctors at their baseline visit and that this same doctor provided the information session. |
| 6 | The intervention was delivered via the internet. Each patient could access the intervention using the unique link on the paper card that was handed to them during their initial visit to the clinic. |
| 7 | The intervention was designed for individual use. Participant recruitment and 4 and 8 week follow up appointments took place at Istanbul Training and Research Hospital Dermatology Department. |
| 8 | Participants were recommended to use topical corticosteroids twice per day for 4 weeks, and moisturizers at least twice per day for 8 weeks. Informational and reminder text messages were sent twice daily for 4 weeks. |
| 9 | The intervention was not tailored to individual users. Text messages were taken from a list of standard prompts (e.g., use soap bars that do not contain colour or perfume’). |
| 10 | None described. |
| 11 & 12 | Compliance to treatment was assessed as the frequency of medication use (at baseline and after 4 and 8 weeks). Compliance to treatment and preventive behaviours were reported: at week 4, 52.9% of the patients in the SMS group and 64.7% of the non-SMS group stated that they forgotten to take, or did not use the treatment, at least once. |
| Hawkins 2016 (46) |  |
| 1 | Web application (app) based education. |
| 2 | Aimed to educate people with psoriasis using videos, digital graphics, and textual information |
| 3 & 4 | A new module was created on the dermatology patient education website, DermPatientEd.com, to display an educational video about psoriasis, text-based information, and graphics about side effects. Each patient received a paper card with a unique link to either the online module and survey (intervention), or just the survey only (control). A research fellow recruited clinic patients to the study. Participants viewed the educational content and completed the survey online. |
| 5 | The intervention is intended for people with psoriasis. No information was provided on the people responsible for creating the website content. Training for intervention providers may not have been applicable as this intervention was delivered online. |
| 6 | The educational intervention was designed for individual use and was delivered remotely via a website. |
| 7 | Participants were recruited from the Wake Forest Baptist Health Dermatology clinic from January 2016 to May 2016. Access to the internet was essential for intervention access. |
| 8 | Participants were asked to browse the information on the website and then complete the survey. |
| 9 | The intervention was not tailored to individual users, only generic information was provided. |
| 10 | No modifications were reported. |
| 11 & 12 | The authors intended to contact participants via telephone who did not complete the survey within 72 hours. They reported distributing 50 paper cards with links to the website or survey (25 per group), and 23 surveys were completed, although one contained an unrecognizable numeric study identifier, leaving 22 valid responses in total. The overall response rate to the survey was 46%. |
| Heckman 2021 (47) |  |
| 1 | An educational website called ITCH-RELIEF (Interactive Toolbox of Comprehensive Health Resources to Enhance Living with Itch – Educational Facilitation). |
| 2 | Aimed to improve itch-related quality of life in adults with atopic dermatitis, psoriasis and chronic itch. |
| 3 & 4 | Participants completed an online baseline survey online before they accessed ITCH-RELIEF, which comprised of five modules addressing the components of the Biopsychosocial Model of Chronic Itch. Intervention providers sent patients reminders via and/or SMS text to use the materials and to rate their scratching pleasure and itch. Patients completed a follow up survey after 4 weeks. |
| 5 | The intervention is intended for people with psoriasis, atopic dermatitis and chronic itch (itch lasting longer than six weeks). No information on the people responsible for creating or delivering the content for the website was provided. Training for intervention providers may not have been applicable because the intervention is delivered online. |
| 6 | The educational intervention was designed for individual use and was delivered remotely via a website. |
| 7 | Access to the internet was a requirement for intervention use. |
| 8 | Participants tested the website over a four-week period. Email and text messages were sent to remind participants to use the materials weekly for 3 weeks and to rate their scratching pleasure and itch. |
| 9 | The intervention content was not tailored to individual users. |
| 10 | No modifications were reported. |
| 11 & 12 | The intervention providers planned to send text or email reminders to patients who had not accessed the website to encourage patients to use the materials and rate their itch and scratching. Website usage was also a secondary outcome. The authors reported 248 individuals completed the screening questionnaire and 173 (69.8%) people were eligible for inclusion. 164 (94.8%) people consented to take part, 137 (83.5%) completed the baseline survey, and 107 (78.1%) completed the follow-up survey and had total length of website visit data recorded. People with atopic dermatitis and psoriasis visited 56.5% of the pages of the website on average. People with atopic dermatitis spent an average of 21.01 (95% CI = 12.6, 29.5) minutes on the 16-page eczema-oriented website. Patients with psoriasis averaged approximately 20.9 (CI = 7.0, 34.7) minutes on the 14-page psoriasis-oriented website. |
| Hedman-Lagerlöf 2021 (48) |  |
| 1 | Internet-delivered Cognitive Behavior Therapy (ICBT). |
| 2 | Rationale: symptoms and distress associated with atopic dermatitis are influenced by learned aversive conditioning, and thus avoidant coping strategies can exacerbate symptoms. Scratching behavior forms part of a vicious cycle in which itch leads to scratching and subsequent skin damage and inflammation. ICBT posits that changing avoidant behaviours is key to reducing symptoms. The aim of the intervention was to reduce symptoms (itch, bleeding, and cracked skin) and improve quality of life, and psychological outcomes (perceived stress, anxiety and insomnia). |
| 3 & 4 | Ten modules covering the following topics: introduction and mindfulness training; CBT model for understanding eczema; protecting the skin barrier; issues with control and avoidance behaviors; conditioned eczema symptoms and exposure; exposure and function of behaviors; exposure to social stigma; continued exposure and value-based action; continued exposure and handling sleep problems (optional); treatment summary; and relapse prevention. Contact between participants and the therapist was made through asynchronous text messages. |
| 5 | The intervention is intended for adults (18+) with atopic dermatitis. Therapists in the study were four licensed psychologists who specialized in CBT. |
| 6 | Therapists delivered ICBT to individuals. The primary role of the therapist was to provide feedback on homework. |
| 7 | The treatment was delivered through a secure and encrypted internet-based treatment platform. |
| 8 | A 12-week programmer including 10 modules to which participants were granted gradual access. |
| 9 | Therapists provided tailored feedback on the participant’s weekly homework assignments and directed the general treatment model to suit the participant’s goals and problems. |
| 10 | No modifications were reported. |
| 11 & 12 | No plans to measure intervention fidelity/adherence outlined, although the number of hours spent using the online intervention and treating patients were reported: Participants receiving ICBT spent on average 10.8 hours reading the text material and 23.6 hours conducting treatment exercises over 12 weeks. ICBT therapists spent on average 39.7 minutes with each participant. |
| Iliffe 2019 (49) |  |
| 1 | A closed Facebook peer support group provided by the charity Alopecia UK. |
| 2 | Rationale: Facebook groups can provide opportunities to share and gain knowledge, emotional support, as well as achieve goals. Online support platforms might offer protection from social stigma and better opportunities for self-expression and facilitate feelings of empowerment. This study aimed to understand the experiences of online support in people living with alopecia. |
| 3 & 4 | A closed group on Facebook for people in the UK with experience of Alopecia. Participants are expected to follow a clear set of rules once accepted into the group. Individuals can post in the group and reply to posts by others. All posts must be approved by charity administration staff before they are published to ensure the culture remains welcoming and friendly. Individuals who break the rules are removed from the group and unsuitable posts are deleted. |
| 5 | The Facebook group is exclusive to people in the UK who have Alopecia and the family members of patients. The group was created by staff from Alopecia UK who also monitor group membership and activity. |
| 6 | A group intervention. Participants require access to the internet and a Facebook account to join the group. Prospective group members are required to answer a set of questions before being allowed to join the group. |
| 7 | The group is delivered via the social media platform Facebook. In addition to internet access, group facilitators are needed to moderate group admissions and activity. |
| 8 | Members of the support group are free to post to the group whenever they wish, providing their post is appropriate and approved by the group monitors. |
| 9 | Tailoring is not applicable to this intervention. Groups members are free to share their personal views and experiences of alopecia, providing the content they share is appropriate and approved by the group monitors. |
| 10 | Group membership and content can be modified by administrative staff from Alopecia UK. |
| 11 & 12 | Group posts are monitored by staff from Alopecia UK to ensure individuals adhere to the rules of the group, to ensure the culture remains welcoming and friendly. Actual intervention adherence/fidelity was not assessed. |
| Joergensen 2020 (50) |  |
| 1 | Electronic memory button and a supportive application (app). |
| 2 | Aimed to determine whether the combination of an electronic memory button and a supportive application (app) would affect the quality of life and subjective and objective severity measures among people with atopic dermatitis over one month following the patient’s normal schedules of treatment. |
| 3 & 4 | The memory button is CE-marked (developed by The HabLab Aps, KlikKit, Copenhagen, Denmark) and connected via Bluetooth to a mobile phone and the corresponding app. Participants tracked treatment use by clicking the button or manually inputting data into the app. The memory button also saves the clicks, which can be added to the app later. Participants attended two consultations with the same doctor 28 ± 3 days apart. Key outcomes were assessed at both consultations. Participants received care and education on medication application based on their preferred topical treatment and were introduced to the button and/or app at the first consultation. |
| 5 | The intervention was designed for people (18+ years) with atopic dermatitis who could read Danish. Doctors were responsible for introducing the button and app, and assessed SCORAD, EASI, POEM, and DLQI at both consultations. |
| 6 | The intervention is designed for individuals and was delivered via a digital memory button and a mobile app. The memory button connects to the mobile app via Bluetooth. |
| 7 | The intervention was tested by researchers in Denmark. The paper does not state where the two consultations were conducted. The memory button/app was used by patients at home or during daily life by people who had an Android phone or an iPhone. |
| 8 | Participants were asked to track their treatment activities, but no further information was provided. It is assumed that participants were required to click a button or register on the app whenever they applied treatment. |
| 9 | Patients were able to choose their preferred topical treatment. |
| 10 | No modifications were reported. |
| 11 & 12 | The authors did not outline plans for assessment of intervention adherence/fidelity. They reported that 84/96 patients completed both consultations, but no additional information was given on those who withdrew from the study. |
| Koulil 2018 (51) |  |
| 1 | Tailored Therapist-guided Internet-based Cognitive Behavioural Treatment (ICBT). |
| 2 | Aimed to provide an in-depth illustration of the course and content of the tailored therapist-guided ICBT, through 2 case reports differing in symptomatology and treatment goals, including challenges and obstacles that might be encountered. Also aimed to investigate the development of the therapeutic alliance in the online treatment. |
| 3 & 4 | ICBT comprised of an introductory module, five treatment modules (pain, itch, fatigue and physical limitations, negative mood, and social relationships), and a closing module. The treatment protocol was based on techniques from standardized protocols for face-to-face treatment of various chronic somatic conditions. One individual with psoriasis attended one or two face-to-face intake sessions with the therapist. A flexible protocol was used for each module and the therapist selected suitable activities and assignments for the person’s goals and clinical profile. Introductory module - treatment goals were set relating to decreasing itch-scratching problems and improving mood. Itch module – psycho-education texts and activities (including habit reversal) plus self-monitoring diary. Negative mood module – stress management (e.g., relaxation exercises, distraction, problem-solving, and reappraising the situation); audio relaxation exercises (progressive muscle relaxation, cued relaxation, and visualization exercise); and relaxation/distraction activities (e.g., reading, gardening, cycling, and walking). Closing module - relapse prevention and further goal attainment. |
| 5 | ICBT was for people with Psoriasis, Psoriatic Arthritis or Rheumatoid Arthritis experiencing anxiety or negative mood. No details given on the therapist who delivered the intervention. |
| 6 | The intervention was delivered to individuals via an online treatment portal. |
| 7 | The intervention was delivered online and access to a computer and the internet were a requirement for participation. |
| 8 | Up to two modules were selected for each patient based on their treatment targets. Duration of modules varied based on participants’ progress through. Activities included goal setting, education, relaxation exercises (once per day), self-monitoring, valued activities, relapse prevention, activity management, sleep hygiene. Feedback on assignments was given once per week. |
| 9 | The ICBT was designed as a tailored, personalized treatment. The therapist selected assignments and online texts that were most suitable to patients’ individual problems, treatment goals, and perpetuating cognitive-behavioural factors, and gave individualized feedback on assignments approximately once a week. |
| 10 | No modifications were reported. |
| 11 & 12 | No plans for assessing intervention adherence/fidelity were outlined. The patients use of the ICBT intervention was reported, including the number of logins, assignments completed, as well as the number of messages sent, and phone calls made to the therapist. The participant logged in 47 times, completed 37 assignments (95% of the total given assignments), received 13 messages from the therapist, and sent 11 messages within a period of 5 months (1-week introduction module, 8-week itch module, 9-week negative mood module, 2-week closing module). One phone call was made by the therapist during the online treatment to clarify a given assignment. |
| Lee 2018 (52) |  |
| 1 | Acceptance and Commitment Therapy Enhanced Behavior Therapy delivered by way of telepsychology. |
| 2 | Aimed to conduct a randomized controlled trial to examine the feasibility of delivering ACT-enhanced behavior therapy as a treatment for trichotillomania by way of telepsychology. |
| 3 & 4 | The intervention protocol closely followed an empirically supported acceptance and commitment therapy-enhanced behavior therapy treatment manual (Woods & Twohig, 2008), which includes traditional habit reversal training techniques and contemporary behavior therapy elements from ACT that employ techniques to change the function of the urges to pull as well as the associated cognitions.  The intervention group completed a pre-treatment assessment and tracked their baseline hair pulling for one week before starting the 12-week treatment. All treatment sessions utilised telepsychology procedures, meaning participants received all treatment in their homes while therapists were located in a private room in a university clinic. A HIPAA approved video conferencing software (VSee) was utilized. All sessions were video and audio recorded and saved to a HIPAA compliant server. All assessments were completed using online survey software (Qualtrics). |
| 5 | The intervention was intended for people with trichotillomania. Advanced graduate students provided treatment under the supervision of a licensed psychologist who co-authored the manual on which the treatment protocol was based. |
| 6 | Intervention is designed for individual users and was delivered via a video conferencing system called VSee. |
| 7 | Participants were based in Utah, USA but received treatment remotely via telepsychology service. Although not stated explicitly, participants required a computer to access the video conferencing software and the internet to complete the required assessments via an online survey software platform called Qualtrics. |
| 8 | Individuals attended 10 one-hour treatment sessions in total, ideally one per week. |
| 9 | The overall length of the intervention was flexible to accommodate the personal schedules of participants. |
| 10 | The paper states that the protocol ‘closely’ followed an empirically supported acceptance and commitment therapy-enhanced behavior therapy treatment manual (Woods & Twohig, 2008), suggesting some modifications may have been made, although it is not clear from the paper if any modifications were made. |
| 11 & 12 | All sessions were video and audio recorded using the built-in recording function in VSee. Twenty percent of all sessions were viewed and scored for the quantity and quality of the coverage of each treatment component using a standardized treatment integrity scoring system used in previous ACT research (Crosby & Twohig, 2016; Twohig & Crosby, 2010). Two sessions from each 10-session treatment were systematically chosen such that each session number was reviewed approximately five times. An independent reviewer scored each of the 48 selected sessions. For every coded session, HRT consistent (i.e., awareness training, competing response training, and contingency management), ACT consistent (i.e., acceptance, defusion, self as context, present moment awareness, values clarification, and committed action), and ACT inconsistent (i.e., cognitive challenging, experiential avoidant change strategies, and thoughts and feelings cause action) behaviors were rated on a five-point Likert-type scale (1 = the variable was never explicitly covered to 5 = the variable occurred with high frequency and was covered in a very in-depth manner). Therapist adherence to the treatment model and therapist competency were rated on the same scale.  Treatment adherence and therapist competency were highly rated. Each process was thoroughly covered in at least one session. On average, the therapists covered acceptance, defusion, and committed action more than other processes, but changes observed in participants from pre to post intervention did not differ significantly between therapists. |
| Manne 2021 (53) |  |
| 1 | MySmartSkin (MSS) web-based intervention. |
| 2 | Aimed to promote skin self-examination and sun protection behaviours. The conceptual framework guiding MSS was the Preventive Health Model (PHM) and prior work evaluating factors associated with skin cancer surveillance and sun protection behaviors among melanoma survivors. |
| 3 & 4 | MSS consisted of an orientation section, a body mole map, and three core sections. Core 1: goals of the intervention, information about melanoma and risks of recurrence, skin cancer risk factors, performing a thorough skin self-examination (SSE); and an overview of sun protection behaviors. Core 2 assessed prior experience with SSE, benefits and barriers to SSE, confidence in conducting skin self-check, and strategies for doing a skin self-check. Core 3 assessed participants’ current sun-safe behaviors guided them to set sun-safety goals and provided a sun-safety action plan. Users could access printable documents from each core section and a summary of the most recent SSE and sun-safe action plan. An online body mole map was provided to record and track moles and other skin growths over time. Participants could log in to use the program to help them complete their monthly SSE. Additional elements: features to promote user engagement and interest (i.e., automated email reminders; tailored content; brief physician videos; quizzes; and interactive game-like activities.  Participants were emailed a unique username and password. They completed a orientation to MSS before gaining access to the core sections. Core sections were access sequentially providing the previous section was completed. |
| 5 | The intervention is designed for individuals diagnosed with stage 0–III melanoma who were 3–24 months post-surgery, had not completed a thorough SSE in the past 2 months, and/or were not adherent to sun protection recommendations, were ≥18 years of age, able to speak and read English, and had access to a computer connected to the Internet. There was no intervention provider. |
| 6 | The intervention is delivered to individuals via the internet. Individuals are emailed a unique username and password to access the online intervention. |
| 7 | The online app intervention was tested by people living in New Jersey, USA but was delivered remotely. |
| 8 | No information on the frequency and duration of the intervention was provided, but the study lasted for 48 weeks. |
| 9 | Tailored activities included selecting reasons why conducting SSE (and engaging in sun protection) is important to the partici.pant, assessing barriers to engaging in SSE (and engaging in sun protection), and completing action plans for SSE and sun protection. |
| 10 | No modifications were reported. |
| 11 & 12 | The intervention was evaluated using three surveys: (a) a 20-item Impact and Effectiveness measure assessing the degree to which MSS helped the participant learn how to be prepared to conduct SSE and engage in sun protection behaviors as well as feel in control of his/her health and feel less worried about melanoma (1 = not at all, 5  = very); (b) a 22-item intervention barriers measure, which evaluated technological barriers (4 items), personal barriers to use (6 items), general barriers to use (6 items), and intervention specific barriers (5 items; 0  =  not a problem, 1 =  a little problem, and 2 =  a major problem); and (c) a 15-item Evaluation and Utility survey, which assessed program characteristics, including usefulness, convenience, ease of use, worry about privacy, ease of navigation, and satisfaction with the program (1 = not at all, 5 = very).  Participant response rate was reported (40.9%). One hundred forty-eight of the 224 participants (66.1%) completed the orientation and all three cores, 25 (11.2%) completed the orientation and two cores, 10 (4.5%) completed the orientation and one core, 19 (8.5%) only completed the orientation core, and 22 (9.8%) did not complete any of the intervention. Almost 38% of participants did not use the Skin Self-check Program to complete SSE, 26.8% used it once, 15.2% used it twice, 7.1% used it three times, and the remaining 12.8% used it between 4 and 12 times over the follow-up. There was less use of the online Sun Safe Action Plan program, with almost 88% not using it and only 4.5% using it more than once. The average number of views that did not involve completing a core module, sun-safe action plan, or skin self-check mole map program was 5.2 (SD = 4.2). |
| Marasca 2020 (54) |  |
| 1 | Psychological video consultations. |
| 2 | Aimed to improve quality of life and psychological well-being. |
| 3 & 4 | The intervention elements and materials were not described, nor were the activities or procedures. |
| 5 | The intervention was intended for people with chronic skin diseases, although no information was provided on the intervention provider. |
| 6 | Individuals attended psychological video consultations. |
| 7 | The psychological video consultations were implemented at the dermatology clinic at the University of Naples Federico II in Italy via the clinic’s teledermatology service. |
| 8 | Each person attended 3 consultations in total. Consultations were held on a bi-weekly basis (every 2 weeks). |
| 9 | No information about tailoring provided. |
| 10 | No modifications were reported. |
| 11 & 12 | The authors did not state any plans for assessing intervention fidelity/adherence and the short report does not provide any information on actual fidelity or adherence to the intervention. |
| Mollerup 2016 (55) |  |
| 1 | Healthy Skin Clinic; a complex nurse-led counselling and website intervention. |
| 2 | The aim of the intervention was to support patient self-management of hand eczema. The aim of the study was to qualitatively evaluate the intervention by exploring the participants’ views of the programme, and quantitatively evaluate intervention usage using data capture from a transaction log. |
| 3 & 4 | The intervention combined face-to-face nurse-led counselling with access to a website that was designed to facilitate self-management. The paper states the intervention was delivered by ‘four trained nurses’ but does not describe the training nurses received.  4) Face-to-face counselling sessions were delivered immediately followed medical consultations. The first counselling session involved a tailored discussion on skin care and an introduction to the website. The second counselling session was tailored the needs of participants based on their personal website profile.  Login to the website was restricted. Each time participants used the intervention, they were required to use two-factor authentication solution, which then prompted a single-use six-digit number. The website had two interfaces: one where patients could communicate and another where patients could communicate asynchronously with the intervention team. It comprised of five menus: (1) patient profile; (2) electronic log for self-monitoring disease; (3) education material and videos; (4) quizzes; (5) patient forum.  As for the electronic self-monitoring log, participants registered the date and rated their disease severity on a scale from 0 to 10. They could note their symptom and reflect on influencing factors/triggers, as well as tick the extent of their eczema on a set of displayed hands in an interactive programme to which photographs could be attached. Participants could refer to the data they inputted, as could the intervention team. Although users were not monitored or contacted regularly by the intervention team. Biweekly emails were sent to participants to prompt use of the website and to contact the team if needed. |
| 5 | The intervention was intended for Danish-speaking adults (18+ years) with hand eczema. The intervention team comprised of four trained nurses and the one academic researcher. |
| 6 | The intervention was intended for individuals, although participants who had access to the website could communicate with other patients via a forum. |
| 7 | Counselling sessions were delivered in-person in the dermatology departments of Gentofte, a metropolitan university hospital, and at a large private dermatology clinic in Aalborg in the northern region of Denmark. Participants needed internet access to use the website. |
| 8 | Two face-to-face counselling sessions; the first at the beginning of the study and the second after individuals had registered on the website. It is not clear how often patients were required to use the online modules. Reminder emails were sent every two weeks. |
| 9 | Counselling sessions were tailored, as participants received personalized recommendations for skin care to suit their lifestyle and the second counselling session was based on information individuals provided in their website profile. |
| 10 | Patients who do not use the internet were given a folder with the same information on pamphlets and additional prints designed for a self-monitoring offline version. |
| 11 & 12 | The intervention team planned to send bi-weekly emails to remind participants to use the website and contact the team if they desired. This relates to attempts to improve intervention adherence. Website use (login frequency) was reported: 37% (52/140) of registered users did not login within the 6-month follow-up. Average login frequency among the 88 people who used the website at least once was 5.1, with a minimum of one visit and a maximum of 20 unique logins. |
| Russell 2019 (56) |  |
| 1 | Online mindfulness-based intervention. |
| 2 | The development of the intervention was informed be a systematic review of mindfulness-based interventions, a survey to understand the knowledge, attitudes and practices associated with meditation among people with melanoma and followed recommendations for adapting mindfulness-based programs. The intervention was aimed to help people with melanoma to understand the potential benefits of using mindfulness in their day-to-day life and to support daily meditation practice. |
| 3 & 4 | A 6-week online mindfulness-based program delivered via a website composed of three main components: (1) an educational component delivered through short videos, (2) formal meditation practices (MP3 audio files) and (3) an informal practice encouraging mindfulness behaviours in daily activities (e.g., “During next week, notice when you are stressed. How is your body responding? What happens to your breathing? What sort of thought activate your stress?”). Each week of the program explored a different topic and built on topics explored in previous weeks. Email reminders encouraging participants to meditate were sent twice daily. including embedded short videos and a downloadable PDF transcript of the videos |
| 5 | The program was designed to be self-managed without any staff or teacher interactions with participants, and allowed for flexible navigation of the website where the content could be accessed according to the user’s preferred order. |
| 6 | The intervention was delivered to individuals online via a website. |
| 7 | Online. Patients were required to have regular access to a digital device (laptop, tablet, smartphone) and received a unique identification number to access the website. |
| 8 | The expected weekly meditation time was 70 min for weeks 1 and 2, and 140 min for weeks 3 to 6. |
| 9 | The content of the intervention was not tailored to individual users, but users were free to navigate the online content in their preferred order. |
| 10 | No modifications were reported. |
| 11 & 12 | Content relevance of the program was recorded weekly through three open-ended questions inquiring about the benefits experienced and aspects of the program liked and disliked. Meditation practice was recorded weekly through self-reported questionnaires capturing the use of any other type of meditation unrelated to the intervention, the frequency and duration of the practice, and, if applicable, reasons for not meditating as recommended. |
| Schuster 2020 (57) |  |
| 1 | Facebook. |
| 2 | Rationale: Some people consider Facebook as an attractive health care tool from which they can retrieve disease-related information and share experiences with similar others, which might support coping. |
| 3 & 4 | Intervention was self-selected access to Facebook. Participants accessed and engaged to suit their needs and preferences. No procedure or protocol for using Facebook was outlined. The aim of this study was to explore the relevance and suitability of Facebook as a source of disease-related information for people with psoriasis. |
| 5 | Facebook is open to the public and so this study did not require an intervention provider, it sought to evaluate an existing platform as a source of disease-related information for people with psoriasis. |
| 6 | Facebook is intended for individual users but provides an opportunity to connect with other users and groups. |
| 7 | Online - Facebook can be accessed through any device that support the internet, or via the existing mobile app. Access to the internet and a user profile is necessary to access Facebook. |
| 8 | The frequency of use was at the discretion of individual users. Facebook use ranged from no access to use several times a day. |
| 9 | Facebook users can create a tailored profile if they wish. |
| 10 | Not applicable to this study. |
| 11 & 12 | Frequency and duration of Facebook use were measured. Frequency: never or less than once per month (n = 25, 25%); at least once per month (n = 5, 5%); at least once per week (n = 12, 12%); Daily (n = 25, 25%); several times daily (n = 34, 34%). Daily average duration: less than 5 min (n = 4, 5%); 5–30 min (n = 43, 57%); 30–60 min (n = 18, 24%); more than 60 min (n = 11, 14%). |
| Sherman 2019 (58) |  |
| 1 | My Changed Body, a web-based self-compassion focused therapeutic writing approach. |
| 2 | Rationale: The intervention has poved efficacious in addressing body image concerns of women in the breast cancer context. (Przezdziecki et al., 2016; Sherman et al., 2018) developed for use with breast cancer survivors. The intervention was adapted for people with visible skin conditions. Aimed to enhance self-compassion and modify emotions, including a decrease in negative affect and increase in positive affect. |
| 3 & 4 | Participants were provided with five specific self-compassion focused prompts to structure their writing based on the concept of self-compassion (Neff & Dahm, 2015), including treating one’s body with kindness, giving kind advice to the self, having connection with others who also experience body image difficulties, awareness of one’s circumstances and reactions in a broader context, and writing a self-compassionate letter to oneself. Participants were instructed to briefly introduce and describe in writing their deepest thoughts and emotions regarding a negative event they had experienced in relation to their condition. |
| 5 | The intervention was intended for people aged 18 years and over experiencing a currently symptomatic and visible skin condition (e.g., eczema, psoriasis, acne) and who had experienced at least one negative event related to their condition (e.g., feelings of failure, humiliation, rejection). No intervention provider. |
| 6 | Intervention was intended for individual users and was delivered via the internet. |
| 7 | Access to the internet was required as the intervention was advertise an delivered online. |
| 8 | Patients were allowed up a maximum of 30 minutes writing time. |
| 9 | The intervention was not tailored to individual users. |
| 10 | No modifications were reported. |
| 11 & 12 | The paper did not outline any strategies for assessing intervention adherence/fidelity. All participants assigned to the intervention (and control) completed the study. |
| Svendsen 2018 (59) |  |
| 1 | ‘MyPso SmarTop’ smartphone app. |
| 2 | Rationale: Topical treatment adherence in psoriasis is often low, which can lead to symptoms worsening. Applications for patient support exist but their role in improving adherence has not been evaluated. Aimed to support adherence to daily application of calcipotriol/ betamethasone dipropionate. |
| 3 & 4 | From week 4 to 26 all patients were provided with Cal/BD cutaneous foam to be used once daily. Participants were asked to replace the canister when empty and were told to bring their medication canisters and dispensers for destruction at the week 4 return visit. They were not told in advance about the use of the data obtained by the electronic monitor or that each medication canister was weighed before/after use until the final week 26. The app provided reminders and information on number of applications/ amounts of foam prescribed. Information was collected from a monitor chip synchronized to the app via Bluetooth. The app ceased to collect data after 28 days. Participants could contact the laboratory assistant via telephone regarding use of the supporting app and electronic monitor. |
| 5 | Intended for people diagnosed with mild to moderate psoriasis. Delivered by investigator and laboratory assistant. |
| 6 | Participants received a canister of Cal/BD cutaneous foam which contained an electronic monitor with a chip registering the day/time used. |
| 7 | The study was carried out at a dermatology outpatient clinic at Odense University Hospital. Patients had to own a smartphone, or skills for using a smartphone provided by investigator (if app not supported by patient’s smartphone operating system). |
| 8 | The app provided once-daily reminders for daily foam application during a 28-day treatment period. Return visits were scheduled for weeks 4, 8 and 26. |
| 9 | Dosage of Cal/Bd cutaneous foam were prescribed for each patient; the appropriate quantity for each application on diseased skin was calculated by determining the involved area expressed as body surface area (BSA) and multiplying by 0.5 g foam per 1% BSA. |
| 10 | If a participant’s smartphone operating system did not support the app, they were provided with a smartphone by the investigator, providing they had the skills to use it. |
| 11 & 12 | Participants could replace the medication canister whenever empty and telephone the laboratory assistant with questions regarding use of the supporting app and electronic monitor. Plans to measure medication adherence involved weighing containers of the topical Cal/Bd cutaneous foam before and after the intervention period. Adherence to medication was reported. Not data on usability of app. |
| Van Beugen 2016 (60) |  |
| 1 | Internet-based Cognitive behavioural therapy (ICBT). |
| 2 | Rationale: People with psoriasis frequently experience a high burden of disease in daily life. Systematic reviews show favourable effects of ICBT for chronic somatic conditions, but research in dermatological conditions is scarce. Aimed to improve physical and psychological functioning and reduce the impact of psoriasis on daily activities. |
| 3 & 4 | The intervention comprised of five flexible treatment modules containing a broad variety of cognitive and behavioural techniques focused on themes that patients often experience problems with: itch, pain, fatigue, negative mood, and social relationships. Participants had face-to-face sessions with a therapist (a psychologist) and discussed personal goals. Researchers informed participants on how to use the website via telephone. Participants had to log in to a secure website which hosted the intervention. Treatment modules/ individual assignments were based on individual goals, therapist’s judgment, and screening procedures. Participants received written feedback on assignments. |
| 5 | The intervention was intended for people with psoriasis aged over 18 years, with a positive psychological risk profile (e.g., impact of chronic skin disease on daily life ISDL score of >5 for anxiety and/or >21 for negative mood). Face-to-face sessions were delivered by a therapist (psychologist). There were six female therapists (mean age =29.67,8.76) who had at least an MSc in Clinical/ Health psychology, previous experience of therapy ranging from 0-7 years (mean=2.17,2.79). Three therapists had completed studies as healthcare psychologists. All therapists were supervised by a senior clinical psychologist trained in CBT. |
| 6 | The ICBT intervention was intended for individuals with psoriasis who accessed the intervention via the internet, by logging on to the secure intervention website. |
| 7 | This intervention was tested on people with psoriasis from outpatient dermatology departments in Dutch hospitals and through the Dutch Psoriasis Association. Participants underwent two initial face-to-face intake sessions with therapist and then logged in to the secure intervention website. |
| 8 | Intervention duration and content varied between participants, depending on treatment goals, with a mean duration of 25 ± 12 weeks (range 1–57 weeks). During the intervention, there was a period of >4 weeks of no contact with the therapist in 20% of patients (N=13) and IBCT was paused for 34% of patients (N=22) because of personal circumstances. IBCT length was calculated excluding the mutually agreed upon weeks of non-intervention. |
| 9 | Participants were given a choice of treatment modules. The assignments within these modules were based on the individual’s goals, therapist’s judgment, and screening procedures. Therapists provided participants with personalized written feedback on their assignments once a week. |
| 10 | No modifications were reported. |
| 11 & 12 | The research team planned to measure adherence to ICBT. Patient satisfaction with ICBT/evaluation of user-friendliness was assessed on a 10-point Likert scale with greater scores indicating greater satisfaction and user-friendliness. Patients were asked to rate to what extent they felt the intervention had a sustained positive effect, and whether they would recommend the intervention to a friend on a 4-point Likert scale ranging from ‘no’ to ‘certainly.’ Intervention attrition was assessed: The ICBT intervention dropout rate was 26.2%: 10 patients did not start treatment (non-starters, 15.4%), 6 patients dropped out during treatment (non-completers, 9.2%), and 1 patient (1.5%) died during treatment. |
| Van Cranenburgh 2015 (61) |  |
| 1 | E-learning quality of life (EQoL): a Web-based, Educational, Quality-of-life Intervention. |
| 2 | Aimed to improve patients’ knowledge and skills to cope with their skin disease more effectively and improve their Health-Related Quality of Life. Intervention components were based on Skindex-29 and content was matched against the stages of change as described in the Transtheoretical Model. |
| 3 & 4 | The website [www.kwaliteitvanleven.nl](http://www.kwaliteitvanleven.nl) consists of 6 components: itch, worries, anger, depression, social contacts and leisure time, which are derived from Skindex-29. After a consultation at the outpatient clinic, participants completed a questionnaire on background characteristics and signed an informed consent form. At the same time, the health care provider completed a questionnaire about the patient. Subsequently, the researcher sent the patient an e-mail with a personal password to log into the website. Patients who had not visited the website during the first 2 weeks, received a reminder by e-mail. If needed, patients could contact a help desk. Eight weeks after inclusion, patients received a questionnaire by postal mail. Patients who did not return the questionnaire within 2 weeks received reminders by e-mail and/or telephone. During participation in the study, patients’ control visits at the dermatology outpatient clinic were planned following routine clinical practice. At the end of the study, health care providers received a questionnaire by postal mail and a reminder by e-mail, if necessary. |
| 5 | The intervention was intended for outpatients (18+) with chronic skin conditions who were recruited by dermatology staff in dermatology clinic. No information about the expertise, background, or training of the people responsible for creating or delivering the content for the website, although researchers were based in dermatology and medical psychology departments. |
| 6 | Individuals accessed the website independently via the internet. |
| 7 | Participants needed to access to a computer with internet connection to access the website. |
| 8 | Participants had access to the website for eight weeks, but no information was provided on the required frequency or duration of use. Individuals who did not visit the website during the first two weeks received an email reminder. |
| 9 | Participants were able to choose parts of the intervention that were relevant to them but the website was not tailored to individual users, nor were e-mails personalised. This was recognised as a limitation and potential improvements. |
| 10 | No modifications were reported. |
| 11 & 12 | Researchers planned to analyse dropouts and participants who were lost to follow-up using independent t-tests, Mann Whitney, and χ2 analyses. Dropouts were reported, plus reasons: 107 eligible and provided consent. One patient did not complete the informed consent form and one patient completed less than 50% of the baseline questionnaire. 105 patients received a website login. Ten patients (9.5%) withdrew before the end of the study (drop-outs), of whom 7 completed the follow-up questionnaire prematurely. Thirty-two patients (31%) did not return the follow-up questionnaires despite reminders. Characteristics of dropouts vs non-dropouts were compared, as were those of people lost to follow up. |
| Zhao 2021 (62) |  |
| 1 | Xingshulin mobile phone patient-to-practitoner system. |
| 2 | Aimed to increase adherence to hospital visits and medication The goal of the study was to investigate the effectiveness of using a mobile phone based messaging app to allow patients to access on demand information about their psoriasis diagnoses and their medications and receive reminders to attend regular clinic check-ins. |
| 3 & 4 | The intervention comprised of a commercially available mobile app called Xingshulin. The app provided medical information, including frequently asked-for information, as well as reminders for application of the drug, reminders for regular visits, and self-assessment forms written in Chinese. The app allowed for communication between participants and certified dermatologists. People with psoriasis and dermatologists participating in the study were required to install the app. Dermatologists received electronic reminders to communicate with patients and were required to respond to patients within 24 hours. |
| 5 | Intended for adults (18+) living with mild to moderate plaque psoriasis who were prescribed Daivobet®. The app provided a remote platform through which patients could communicate with certified dermatologists asynchronously, and so the app was also intended to be used by health professionals specialized in dermatology. |
| 6 | The app was designed for individual users and treatment information and reminders were delivered remotely via the app. Participants received a prescription for Daivobet® in person and were expected to attend clinic appointments at weeks 2, 8, 16, 28, 48, and 52 of the study. |
| 7 | The mobile-based intervention was tested with people attending twelve hospitals in China. The content of the app was written in Chinese. Access to a smartphone was needed to download and use the app, although this was not stated in the eligibility criteria. It was unclear if participants used their personal smartphones or where given one to use for the duration of the study. |
| 8 | People with psoriasis received a 1-year treatment protocol for Daivobet®. Participants were prescribed Daivobet® once daily use for 2–4 weeks to flatten their skin lesions, after which they were to use Daivobet® once every other day until the colour of their lesions faded. Daivobet® was then prescribed for use twice per week, or Devonex® once daily for at least eight weeks, for skin maintenance before individuals switched to ’treat-as-needed’ until week 52. The app sent automatic messgaes to dermatologists reminding them to inquire with patients (twice in the first 4 weeks, and once in 4 weeks after the second month). It is unclear how often people with psoriasis were required to use the app. |
| 9 | People with psoriasis were able to use the treatment as needed for part of the study. It was not clear whether the messages sent to patients were generic or tailored. |
| 10 | No modifications were reported. |
| 11 & 12 | In terms of medication adherence, 41/221 participants returned their medication adherence questionnaire in week 12. Visit rates in the intervention and control group were reported. The investigators stated that they were not able to track the reasons why participants did not complete the study, nor could they record the frequency or duration of app use. |
| 1: Brief name – name or phrase used to define the intervention.  2: Why – rationale, theory or goal of the intervention.  3: What (materials) – description of the elements of the intervention, including materials, and (if applicable) training for intervention providers.  4: What (procedures) – description of intervention activities and delivery procedure(s).  5: Who – information about the intervention provider (title, expertise, background and if training was given) and target audience.  6: How – mode of intervention delivery, including whether the intervention is delivered to individuals or a group.  7: Where – name location or setting where the intervention was delivered and infrastructure required to facilitate its delivery.  8: When/how much – intervention frequency and duration of the intervention, including sessions/activities that form part of the intervention.  9: Tailoring – information about how the intervention was personalized (what, why, when and how).  10: Modifications – description of any changes made to the intervention during the study (what, why, when and how).  11: How well (planned) – plan for assess and/ or improve intervention adherence/ fidelity.  12: How well (actual) – degree to which the intervention was delivered as intended (only if intervention adherence/fidelity was assessed). | |
|  | |

# Outcome variables and results of intervention effectiveness by study

| Outcome | Results |
| --- | --- |
| Alinia 2017 (40) |  |
| Alcohol consumption/  smoking status | When these covariates were included in the analyses, the significant between-group difference in PASI scores at month 12 no longer reached statistical significance. |
| Treatment adherence | Significantly higher in the intervention group relative to controls from month one to ten, but rates declined over time. |
| Disease severity | Significant improvement in PASI scores at months one and three. No significant change in IGA at month 1, 3 or 12. |
| Armstrong 2011 (41) |  |
| Knowledge of condition | Significant improvement after 12 weeks. |
| Disease severity | Significant improvement in POEM scores in the intervention group at 3 months, compared to controls. |
| Satisfaction with intervention | (appeal and usefulness) |
| Balato 2013 (42) |  |
| Quality of life | Significant between-group difference favouring the intervention group after 12 weeks. |
| Treatment adherence | No significant between-group difference in the number of days per week participants forgot to take their medication. |
| Disease severity | Significant improvement in PASI, SAPASI, BSA, GPA scores at three months. |
| Patient-physician relationship | The intervention group reported an improvement after 12 weeks, which was not observed in the control group. |
| Bundy 2013 (43) |  |
| Beliefs about condition | Results not reported. |
| Anxiety | Significant improvement observed for the intervention group from baseline to six month follow up, compared to controls (complete case analysis). |
| Depression | No significant difference. |
| Quality of life | Significant between-group difference favouring the intervention group after six weeks. |
| Alcohol consumption | Results not reported. |
| Smoking status | Predicted missing outcomes. |
| Disease severity | No significant between-group difference in self-reported psoriasis severity at 6 months. |
| Domogalla 2021 (44) |  |
| Mood | Significant improvement observed in the intervention and control group. Group membership had no significant effect. |
| Quality of life | Improvement in QoL but change not statistically significant. |
| Anxiety | Improvements seen in all patients at weeks 12 and 24. The difference in group anxiety scores at week 60 did not reach the threshold of statistical significance. Lower levels of anxiety remained stable at weeks 36 and 60 in people who used the app less than 20% (once every 5 weeks). |
| Depression | Significant reduction observed in all patients from baseline to week 60. The intervention group showed a significantly greater reduction in depression scores at weeks 12 and 24. |
| Alcohol consumption/smoking status | Reported for demographic purposes only. |
| Daily activities | Reduction in impairment on daily activities in all participants, but the difference between the intervention and control group was not significant. |
| Disease severity, itch and pain | Reductions in PASI, itch and pain were seen in both groups at week 60. Differences between groups were not significant. |
| Erdil 2020 (45) |  |
| Knowledge of condition | Significant improvement after 8 weeks. |
| Protective behaviours | The intervention group showed a significant increase in moisturiser use from baseline to week 4 compared to controls. No differences in hand washing or the use of gloves, liquid soap, cologne, or wet wipes were observed. |
| Treatment compliance | The difference in the percentage of people in the intervention (52.9%) and control (64.7%) groups who forgot to take, or did not use their medication, was not significant. |
| Disease severity | Significant reduction in HECSI scores after two months. |
| Hawkins 2017 (46) |  |
| Knowledge of condition | Significant and immediate improvement. |
| Concerns | Seven participants reported not all their concerns were addressed by their clinician. |
| Treatment adherence | Participants were not more likely to take their medication as prescribed post-intervention. |
| Satisfaction with intervention | - |
| Heckman 2021 (47) |  |
| Itch cognitions | There was a significant difference in catastrophizing, but not coping, cognitions from baseline to follow up. |
| Perceived Efﬁcacy in Patient-Physician Interactions | Result not reported. |
| Stress | Result not reported. |
| Itch-related quality of life | Significant within-group differences in QoL after four weeks, also for the emotion subscale. |
| Scratch | A significant reduction in scratch intensity and especially scratch impact was observed after one month relative to baseline scores. Significant difference between baseline and follow up scores on the sleep-related itch and scratch scale, suggesting the intervention group experienced a reduction in scratching during the night. |
| Risk of treatment non-adherence | Result not reported. |
| Itch | Significant improvement in itch severity at 1 month. |
| Frequency and duration of intervention use | - |
| Completion rate | - |
| Hedman-Lagerlöf 2021 (48) |  |
| Stress | Significantly larger reduction in perceived stress in intervention group compared to control group. |
| Anxiety | No significant between-group difference. |
| Depression | Significantly larger reduction in depression scores in the intervention group compared to controls. |
| Quality of life | Significant between-group difference in QoL when using the BBQ scale, but not the Dermatology Life Quality Index. |
| Sleep problems | Compared to the control group, participants in the intervention group reported a significant reduction in sleep problems from baseline to six month follow up, which remained stable after 12 months. |
| Itch intensity (past 48 hours) | Secondary analyses indicated a significant improvement in itch intensity from baseline to follow up. |
| Disease severity | Moderate to large reduction in symptoms (POEM), which was sustained at 12 month follow up. |
| Self-rated health status | Mean scores remained the same from baseline to post treatment, but increased from post-treatment to 6 month follow up and returned to original mean score after 12 months. |
| Satisfaction with intervention | - |
| Iliffe 2019 (49) |  |
| Personal experiences of an online support group for people living with alopecia | - |
| Joergensen 2020 (50) |  |
| Quality of life | No significant between-group difference. |
| Treatment adherence | Used proxy measures of adherence and interpreted the results as ‘improved adherence.’ |
| Disease severity | Significant improvement in POEM, EASI and SCORAD scores observed in the intervention group compared to controls. |
| Koulil 2018 (51) |  |
| Illness cognitions | Significant improvement in helplessness but not acceptance. |
| Worry | No significant improvement found from baseline to post-intervention or follow up. |
| Anxiety | Improvement of at least 30% post-intervention that was not sustained at follow up; slight increase in anxiety scores after six months. |
| Depression and negative mood | Improvements of at least 30% post-intervention and after six months. |
| Sleep hygiene | Result not reported. |
| Scratch | Significant reduction observed. |
| Treatment compliance  (frequency of medication use) | No change in maximal compliance throughout the study. |
| Disease severity | Despite patient-reported improvements, clinician-assessed disease severity worsened slightly from baseline and six month follow up. |
| Patient-physician relationship | Scores obtained for face-to-face sessions were either maintained or improved following the online intervention, but it is not clear whether any of the changes observed were statistically significant. |
| How useful/motivating patient found contact with the therapist | - |
| Satisfaction with intervention | - |
| Daily activities (role limitations) | The individual with psoriasis reported maximal impact at the beginning of the study. No change from baseline to post-treatment or follow up. |
| Perceived social support and stigmatization | High levels of social support and low levels of stigma at baseline were maintained over the study period. |
| Lee 2018 (52) |  |
| Shame | Scores reduced from pre to post treatment for all participants, but the difference between groups was not significant. The combined analysis showed a significant reduction (18.2%) in scores from pre-treatment to follow up. |
| Quality of life | Improvement in QoL but change not statistically significant. |
| Psychological inflexibility | Scores decreased in the intervention (19.6%) and control (3.4%) groups pre to post intervention, but the difference was not significant, nor were the combined scores from post intervention to follow up. |
| Hair pulling | Significant difference between intervention (reduced by 42.2%) and control (increased by 17.7%) group. The combined analysis revealed a significant reduction (39.4%) from pre to post treatment. Scores decreased (26.3%) from post treatment to three month follow up, but the change was not statistically significant. |
| Patient-physician relationship | Participants reported high average scores. |
| Satisfaction with intervention | - |
| Manne 2021 (53) |  |
| Knowledge of condition | Partially and in-directly mediated the relationship between intervention use and performing skin self-examination and sun protection behaviours. |
| Self-efficacy for performing a skin self-examination | Partially and in-directly mediated the relationship between intervention use and performing skin self-examination. |
| Self-efficacy for performing sun protection behaviours | Partially mediated (29%) the relationship between intervention use and sun protection behaviours. |
| Performance of sun protection behaviours (use of sunscreen, hats, long-sleeves, and staying in the shade). | Intervention group reported performing more sun protection behaviours at 24 weeks compared to the control group, although the effect was small. This effect was also evident at 48 week follow up when covariates were included in the analysis. |
| Performance of a skin self-examination | Significant difference between the number of participants reporting performing a skin self-examination at baseline and 24 and 48 week follow up. |
| Number of page visits | - |
| Completion rate | - |
| Barriers (technological, personal and general barriers to use, and intervention-specific barriers) | - |
| Perceived effectiveness of intervention | - |
| Programme characteristics (usefulness, convenience, ease of use and navigation, worry about privacy, and satisfaction) | - |
| Marasca 2020 (54) |  |
| Quality of life | Significant within-group differences in QoL after four weeks. |
| Psychological well-being | Improvements were observed from baseline to weeks two and four, but the changes were not statistically significant. |
| Mollerup 2016 (55) |  |
| Self-efficacy | Intervention group showed a greater improvement in confidence for self-managing eczema compared to controls, but the difference was not statistically significant. |
| Quality of life | Significant between-group difference favouring the intervention group after 6 months.(55) |
| Treatment adherence | No significant difference between website users and non-website users regarding self-reported medication adherence. |
| Itch (in the past 4 weeks) | Significant improvement at 6 month for intervention group. |
| Disease severity | No significant difference in hand eczema severity (HECSI) at 6 months. |
| Number of page visits | - |
| Protective behaviours | Significant improvement in performing protective habits (e.g., use of topical steroids and consulting GP) observed in the intervention group compared to controls. |
| Schuster 2020 (57) |  |
| Affect | No significant differences were found between Facebook users and non-users in terms positive or negative affect. |
| Happiness | No significant differences were found between Facebook users and non-users in terms of happiness. Higher levels of Facebook envy were associated with lower levels of happiness. |
| Facebook envy | Higher levels of Facebook envy tended to report lower levels of subjective well-being and happiness. |
| Quality of life | Higher DLQI was associated with more frequent searching for disease-related information on Facebook. |
| Life satisfaction | No significant differences were found between Facebook users and non-users in terms of life satifaction. Higher levels of Facebook envy were associated with lower levels of life satisfaction. |
| Frequency and duration of intervention use | No association between Facebook envy, happiness or subjective well-being and Facebook use or habits (e.g., searching for disease information). |
| Sherman 2019 (58) |  |
| Affect | Significant and immediate improvement in negative, but not positive affect, compared to controls. |
| Body image related distress | No significant effect on self-compassion or positive or negative affect when analysed as a covariate. |
| Self-compassion | A significant and immediate improvement was observed in the intervention group, compared to the control group. |
| Perceived disease severity | Analysed as a covariate but did not affect the significant differences observed in self-compassion and negative affect. |
| Svendsen 2018 (59) |  |
| Quality of life | Significant between-group difference favouring the intervention group after eight weeks. QoL scores decreased 26 weeks. |
| Smoking status | Included as a covariate but result not reported. |
| Treatment adherence | Self-reported adherence was higher than that as measured by the weight of medication canisters, and the intervention group indicated greater adherence to the cutaneous foam compared to the control group, but the differences were not statistically significant. Significant between-group difference in treatment adherence as measured by electronic medication dispenser. |
| Disease severity | The intervention group showed a significantly greater reduction in LS-PGA compared to the control group at week 4. Similar effects were seen at weeks 8 and 26, although these were not statistically significant. |
| Patient-physician relationship | Positive perceptions of the therapeutic alliance pre-intervention were correlated with greater improvements in psoriasis outcomes post-intervention. |
| Russell 2019 (56) |  |
| Rumination | Reduced rumination related to melanoma, but not statistically significant. |
| Stress and worry | No significant between-group difference. |
| Fear of cancer recurrence | Significant reduction in the severity of fear of cancer recurrence at follow up compared to controls, but few scores fell below the clinical cut-off (≥ 13). |
| Experience of mindfulness | Nineteen participants reported having had some experience with meditation. |
| Frequency of informal meditation practice | The average time spent meditating per week varied from 64 minutes in week 2 to 129 minutes in week 5. |
| Relevance of intervention content (benefits and participant preferences) | - |
| Perceived helpfulness of intervention | - |
| van Beugen 2016 (60) |  |
| Treatment compliance | No significant between-group difference. |
| Psychological functioning (depression, negative mood and anxiety) | No significant between-group differences were found for anxiety, depression, negative mood, or psychological functioning overall. |
| Disease severity | No significant between-group difference. |
| Physical functioning | After six months, the intervention group showed significantly larger improvements compared to the control group. Significant effects were found for fatigue but not for itch. |
| Role limitations | After six months, a significant improvement was observed for role limitations due to emotional and physical health problems in the intervention group compared to the control group. The improvement in role limitations due to emotional problems was further enhanced at follow up. The secondary analysis, which included baseline values of the dependent variable, showed no significant between-group difference. |
| van Cranenburgh 2015 (61) |  |
| Frequency and duration of use | - |
| Number of page visits | - |
| Factors preventing intervention use | - |
| Relevance of intervention | - |
| Convenience of intervention | - |
| Intervention design  (layout and attractiveness) | - |
| Intervention design  (font size and text length) | - |
| Zhao 2020 (62) |  |
| Treatment adherence | Thirteen (31.7%) out of 41 participants who completed the 12-week follow up questionnaire reported using the drug ‘sometimes’ or ‘never.’ |
| Visit adherence | No significant between-group difference. |

# Digital intervention usage

Participants with atopic dermatitis and psoriasis who received the ITCH RELIEF (47) website visited the website three times on average and accessed 56.5% of the website pages over the study period. Participants spent on average 21.01 and 20.90 minutes (respectively) on the condition-specific areas of the website. Participants with atopic dermatitis spent an average of 21.01 (95% CI = 12.6, 29.5) minutes on the 16-page eczema-oriented website. Participants with psoriasis averaged about 20.9 (CI = 7.0, 34.7) minutes on the 14-page psoriasis-oriented website. (47)

In a study with people with melanoma, (53) approximately 66% of participants completed the orientation and all three core modules, and just under 10% of participants reported not completing any of the intervention. Older age was found to be the only predictor of more intervention use. Almost 38% of participants did not use the Skin Self-check Program to complete a skin self-examination and approximately 27% used it only once. Use of the online Sun Safe Action Plan program was poor; almost 88% did not use it at all and only 4.5% used it more than once. (53)

One study (55) recorded how frequently participants visited pages of a website, including tailored user profiles, disease activity monitoring and an asynchronous patient forum, that was delivered in conjunction with face-to-face counselling sessions. Many participants (76%) accessed more than one information page via the information site menu and the patient dialogue forum (72%). Over half (55%) of the website users took one or more quizzes.

Another study (61) found 83% of website users visited the website one or more times. Patients viewed homework/assignments (778 views, 18%), self-assessments (355 views, 8%), and interviews with patients (213 views, 5%) most often. Ten percent of patients experienced technical problems (i.e., login and printing).

People with psoriasis who received an educational programme on psoriasis and a smartphone app for monitoring psoriasis, showed significant reductions in depression and anxiety at weeks 12 and 24, which also persisted long term for participants who used the app less than 20% (once every five weeks); lower anxiety were evident at weeks 36 and 60, and reduced depression scores remained at week 60. (44)
